# Effects of Atmospheric Pressure Plasmas on Isolated and Cellular DNA—A Review

**DOI:** 10.3390/ijms16022971

**Published:** 2015-01-29

**Authors:** Krishna Priya Arjunan, Virender K. Sharma, Sylwia Ptasinska

**Affiliations:** 1 Radiation Laboratory, University of Notre Dame, Notre Dame, IN 46556, USA; E-Mail: karjunan@nd.edu; 2 Department of Environmental and Occupational Health, School of Public Health, Texas A&M University, 1266 TAMU, College Station, TX 77843, USA; E-Mail: vsharma@sph.tamhsc.edu; 3 Department of Physics, University of Notre Dame, Notre Dame, IN 46556, USA; 4 Harper Cancer Research Institute, University of Notre Dame, Notre Dame, IN 46556, USA

**Keywords:** plasma medicine, atmospheric pressure plasma, DNA, ROS/RNS, ATM/ATR

## Abstract

Atmospheric Pressure Plasma (APP) is being used widely in a variety of biomedical applications. Extensive research in the field of plasma medicine has shown the induction of DNA damage by APP in a dose-dependent manner in both prokaryotic and eukaryotic systems. Recent evidence suggests that APP-induced DNA damage shows potential benefits in many applications, such as sterilization and cancer therapy. However, in several other applications, such as wound healing and dentistry, DNA damage can be detrimental. This review reports on the extensive investigations devoted to APP interactions with DNA, with an emphasis on the critical role of reactive species in plasma-induced damage to DNA. The review consists of three main sections dedicated to fundamental knowledge of the interactions of reactive oxygen species (ROS)/reactive nitrogen species (RNS) with DNA and its components, as well as the effects of APP on isolated and cellular DNA in prokaryotes and eukaryotes.

## 1. Introduction

The nascent field of plasma medicine is a rapidly growing and innovative interdisciplinary endeavor encompassing plasma physics, life sciences, biochemistry, engineering and clinical medicine [1]. Electrical plasma ignited in gas under ambient conditions, called an “atmospheric pressure plasma” (APP), is an ionized gas composed of charged particles (electrons, positive and negative ions), radicals, neutral species (excited atoms and molecules), photons (visible and UV) and electromagnetic fields. An important feature of non-equilibrium (cold) APP is its ability to produce a mixture of biologically active agents, such as reactive oxygen species (ROS) and reactive nitrogen species (RNS), while remaining close to ambient temperature, which enables its safe application to living cells and tissues. The physical and chemical properties of the APP, and thus, the formation of plasma products, can be modified by using different types of APP (e.g., APP jets (APPJs), dielectric barrier discharge (DBD)), various configurations of plasma sources, or by varying the voltage applied, type of feed gas and its flow rate [2,3,4]. Thus, the type and dose of reactive species, as well as their distribution and penetration into the tissue, can be readily controlled.

**Figure 1:**
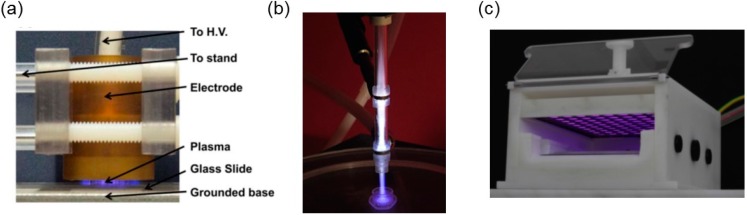
Photograph of various Atmospheric Pressure Plasma (APP) sources in operation: (**a**) a direct floating electrode-dielectric barrier discharge (FE-DBD) in ambient air (adapted from [7], 2011); (**b**) an indirect APP jet (APPJ) ignited in helium (adapted from [45] with permission from Elsevier, Inc., 2014); and (**c**) a hybrid FlatPlaSter in ambient air (reprinted from [15] with permission from Elsevier, Inc., 2013).

One of the APPs widely used for the direct treatment of cells and tissues is a DBD ignited in ambient air [5,6,7,8,9]. The DBD is also known as a “silent discharge” and typically consists of two electrodes, one connected to a high voltage and the other grounded, with either one or both of the electrodes covered with a dielectric material [10]. While DBD plasma provides the delivery of high concentrations of ROS/RNS directly to the treatment material, it is unable to treat non-homogenous surfaces. The floating electrode-DBD (FE-DBD) developed by Fridman *et al.* [6,9,11] for *in vivo* applications has the dielectric material covering the high-voltage electrode, while the tissue acts as the ground electrode. This configuration greatly reduces the flow of current to the treatment tissue. Another commonly used APP is APPJ, which is an indirect source since the plasma generated between two electrodes is transported to the treatment material using a feed gas, typically helium, argon or nitrogen [12,13,14]. The concentration of ROS/RNS reaching the treatment material is typically lower than that obtained with direct DBD. APPJ offers the advantage of treating irregular surfaces and oddly shaped objects. In addition to the above-mentioned direct and indirect APP sources, Isbary *et al.* [15,16] developed several hybrid plasma sources that provide the advantages of both direct and indirect APPs. Two such hybrid sources include FlatPlaSter and MiniFlatPlaSter, which are based on a surface microdischarge (SMD) technology. The SMD technology, in which a dielectric material is sandwiched between a high-voltage and a ground wire mesh electrode, has the advantage of generating a homogenous plasma discharge in atmospheric air without the need for special voltage requirements [15,16]. The hybrid sources allow direct treatment of living objects while eliminating the risk of current flowing through it. Typical DBD, APPJ and hybrid sources are shown in Figure 1, and their production and applications have been reviewed in detail by [1,4,17].

Over the last decade, APPs have shown great potential in a multitude of biomedical applications, including inactivation of bacteria, fungi, viruses and spores [16,18,19,20,21], sterilization of wounds and surgical instruments [6,22,23,24,25,26,27], tissue scaffold treatment [28], cell transfection [29,30], dentistry [31,32], and apoptosis induction in cancer cells [11,33,34,35,36,37,38]. Of the various factors produced by plasma, ROS/RNS have been implicated in having a crucial role in many of these applications. Interestingly, ROS/RNS, in low levels, play an important role in vital physiological processes. Low doses of ROS/RNS have been shown to promote cell survival, proliferation and migration, while excessive ROS levels leading to oxidative stress have been associated with cell senescence [39,40], and the initiation and execution of apoptosis [41,42]. Extensive research has shown that these cellular responses can be initiated by severe oxidative DNA damage [43,44].

Several studies have attempted to characterize DNA damage and the associated cellular responses induced by APPs (Table 1). In this review, we briefly describe the various ROS/RNS involved in DNA damage. The DNA damage response and repair mechanisms in eukaryotic systems pertaining to oxidative stress are also summarized. Further, the effects induced on isolated and cellular DNA by the interactions of ROS/RNS present and/or produced in biological systems due to APP treatment are outlined in detail.

**Table 1:** Summary of various types of APPs and feed gases used to characterize the effect of APPs on isolated and cellular DNA.

Author	Year	Type of APP	Feed Gas	Reference
Leduc *et al.*	2009, 2010	Plasma jet	He	[29,46]
Alkawareek *et al.*	2014	Plasma jet	99.5% He/0.5% O_2_	[45]
Bahnev *et al.*	2014	Plasma jet	He	[47]
Han *et al.*	2014	Plasma jet	He	[12]
Hosseinzadeh Colagar *et al.*	2013	Plasma jet	99% Ar/1% Air	[48]
Kim *et al.*	2006	Plasma jet	He/O_2_	[49]
Li *et al.*	2008	Plasma jet	He	[50]
Niemi *et al.*	2012	Plasma jet	He/O_2_	[51]
O’Connell *et al.*	2011	Plasma jet	He/0.5% O_2_	[52]
Ptasinska *et al.*	2010	Plasma jet	He	[53]
Stypczyńska *et al.*	2010	Plasma jet	He	[54]
Yan *et al.*	2009	Plasma jet	He/1% O_2_	[55]
Young Kim *et al.*	2012	Plasma jet	He/O_2_	[56]
Lackmann *et al.*	2012, 2013	Plasma jet	He/0.6% O_2_	[57,58]
Antoniu *et al.*	2012	Plasma jet	He	[59]
Kurita *et al.*	2011, 2014	Plasma jet	Ar	[60,61]
Sousa *et al.*	2010, 2012	Micro cathode sustained discharge (MCSD) array	He/O_2_/NO	[62,63]
Blackert *et al.*	2013	DBD	Ambient air	[8]
Brun *et al.*	2011	Plasma jet	He	[64]
Chang *et al.*	2014	Plasma jet “torch with spray type”	He/O_2_	[65]
Han *et al.*	2013, 2014	Plasma jet	N_2_	[13,66]
Isbary *et al.*	2013	SMD	Ambient air	[15]
Kalghatgi *et al.*	2010, 2011, 2012	DBD	Ambient air	[7,67,68]
Kim *et al.*	2010	Surface type APP	Ambient air	[69]
Kim *et al.*	2011	Plasma jet with micronozzle array	N_2_	[70]
Ma *et al.*	2014	DBD	He	[71]
Morales-Ramirez *et al.*	2013	Plasma needle	He	[72]
Plewa *et al.*	2014	Plasma jet	He	[73]
Vandamme *et al.*	2011	FE-DBD	Ambient air	[33]
Volotskova *et al.*	2012	Plasma jet	He	[74]
Wende *et al.*	2014	Plasma jet	Ar	[14]
Yan *et al.*	2010	Plasma jet	He	[75]
Choi *et al.*	2012	Microwave plasma	Ar	[76]
Lazovic *et al.*	2014	Plasma needle	He	[77]
Wu *et al.*	2013	FE-DBD	Ambient air	[9]
Lee *et al.*	2014	Plasma jet	N_2_/air	[78]
Ryu *et al.*	2013	Plasma jet	Ar	[79]
Joshi *et al.*	2011	DBD	Ambient air	[80]
Kvam *et al.*	2012	FE-DBD	Ambient air	[81]
Tseng *et al.*	2012	Plasma jet	He/N_2_	[82]
Mols *et al.*	2013	Plasma jet	N_2_	[83]
Sharma *et al.*	2009	Plasma jet	Ar	[84]
Winter *et al.*	2011	DBD	Ar	[85]
Lu *et al.*	2014	DBD	Air, 90% N_2_/10% O_2_, and 65% O_2_/30% CO_2_/5% N_2_	[86]
Venezia *et al.*	2008	Plasma glow	1% ethylene/50% O_2_/49% N_2_	[87]
Yasuda *et al.*	2010	DBD	Ambient air	[88]
Wang *et al.*	2010	Plasma jet	He	[89]
Fang *et al.*	2013	Plasma jet	He	[90]

## 2. Reactive Species Involved in DNA Damage

The reactive species that participate in the degradation of DNA include both free radicals and non-radical species (Table 2) [91]. Some of the common ROS include hydrogen peroxide (H_2_O_2_), ozone (O_3_), superoxide anion (O_2_^●−^), hydroperoxyl (HO_2_^●^), alkoxyl (RO^●^), peroxyl (ROO^●^), singlet oxygen (^1^O_2_), hydroxyl radical (^●^OH), and carbonate anion radical (CO_3_^●−^). Meanwhile, some of the RNS include nitric oxide (^●^NO), nitrogen dioxide radical (^●^NO_2_), peroxynitrite (ONOO^−^), peroxynitrous acid (OONOH), and alkylperoxynitrite (ROONO). ROS and RNS are interconnected and cause DNA damage in biological processes [92]. An example of reactions involving ROS and RNS is given below.

**Table 2:** A list of various reactive species.

Free Radicals	Non-Radicals
**Reactive Oxygen Species (ROS)**	
Superoxide, O_2_^●−^	Hydrogen peroxide, H_2_O_2_
Hydroxyl, ^●^OH	Ozone, O_3_
Hydroperoxyl, HO_2_^●^ (protonated superoxide)	Singlet, ^1^O_2_
Carbonate, CO_3_^●−^	Organic peroxides, ROOH
Alkoxyl, RO^●^	Peroxynitrite, ONOO^−^
Peroxyl, RO_2_^●^	Nitrosoperoxycarbonate, ONOOCO_2_
Carbon dioxide radical, CO_2_^●−^	
**Reactive Nitrogen Species (RNS)**	
Nitric oxide, ^●^NO	Nitrous acid, HNO_2_
Nitrogen dioxide, ^●^NO_2_	Peroxynitrite, ONOO^−^
	Peroxynitrous acid, ONOOH
	Alkyl peroxynitrites, ROONO
	Alkyl peroxynitrates, RO_2_ONO

Nitric oxide and superoxide radical anions can combine to yield peroxynitrite Equation (1) [93]. At neutral pH, ONOO^−^ exists in equilibrium with the unstable ONOOH (p*K*_a_ = 6.5–6.8), which gives ^●^OH and ^●^NO_2_ radicals with a yield of *x* ~0.30, as represented in Equation (2).

^●^NO + O_2_^●−^ → ONOO^−^*k* = 6.7 × 10^9^ M^−1^·s^−1^ (1)

ONOOH → *x* (^●^NO_2_ + ^●^OH) + (1 − *x*) (NO_3_^−^ + H^+^) *k* = 1.3 s^−1^ (2)

The lifetime of ONOO^−^ in buffered carbonate buffer is decreased due to its reaction with free carbon dioxide (CO_2_), which results in a highly unstable nitrosoperoxycarbonate (ONOOCO_2_^−^) anion Equation (3). The ONOOCO_2_^−^ then decomposes into ^●^NO_2_ and CO_3_^−●^ radicals (yield, *y* = 0.33) Equation (4).

ONOO^−^ + CO_2_ → ONOOCO_2_^−^ (3)

ONOOCO_2_^−^ → *y* (^●^NO_2_ + CO_3_^●−^) + (1 − *y*) (NO_3_^−^ + CO_2_) (4)

The high levels of bicarbonate in interstitial (30 mM) and intracellular (12 mM) fluids suggest that the reaction between ONOO^−^ and CO_2_ is the major pathway of decay of peroxynitrite in biological systems [94,95].

The redox potentials of some of the ROS and RNS are given in Table 3. Among ROS, ^●^OH with a redox potential of 1.89 V *vs.* the potential of normal hydrogen electrode (NHE) is a strong oxidant. The ^●^OH has the ability to abstract the hydrogen atom from the C–H bond. The ^●^OH can also be added to C=C bonds at a faster rate than that for hydrogen abstraction [96]. Carbonate radical anions (CO_3_^●−^) with a redox potential of 1.59 V *vs.* NHE can oxidize biomolecules selectively by one-electron abstraction mechanisms [97]. In comparison, ^●^NO_2_ is a milder oxidant. The redox potentials of the DNA bases are 1.7, 1.6, 1.42, and 1.29 V for thymine (T), cytosine (C), adenine (A), and guanine (G), respectively [98,99]. Among these radicals, ^●^OH, CO_3_^●−^, and ^●^NO_2_ are capable of damaging biomolecules, and show different reactivity towards DNA residues and DNA itself.

DNA is composed of two polynucleotide strands wound around each other to form a three-dimensional double-helix structure. Each nucleotide is, in turn, comprised of a five-carbon (deoxyribose) sugar, a phosphate group and a nitrogenous base. The nucleotides in each strand are covalently linked by the phosphodiester bond between the sugar and phosphate molecules, thus forming the sugar-phosphate backbone of the DNA strand. There are two basic categories of bases: the purines (adenine and guanine) and the pyrimidines (thymine and cytosine). The base is attached to the deoxyribose via the *N*-glycosidic bond. The two antiparallel strands of the DNA are held together by hydrogen bonds between the complementary base pairs, A-T and G-C. With regards to the hydrolytic stability of the various bonds in DNA, the most labile under physiological conditions is the *N*-glycosidic bond. Any modification to DNA nucleobases such as oxidation by ROS/RNS can hydrolyze the *N*-glycosidic bond, thus separating the nucleobase from the deoxyribose leaving an apurinic/apyrimidinic (AP) site.

The fundamental chemistry and radical generation, as well as usual reactivity trends with DNA and its components [100], and with amino acids, peptides and proteins [101], have been summarized previously. Both the deoxyribose sugar and the nucleobases of DNA are susceptible to direct oxidative/nitrosative attacks by ROS/RNS. Under physiological conditions, O_2_^●−^ and H_2_O_2_ appear incapable of directly causing strand breaks or nucleobase modifications in DNA [100,102]. However, treatment of mammalian cells with H_2_O_2_ has been reported to induce DNA strand breakage, which is abrogated in the presence of ^●^OH scavengers [103]. Hence, it appears that the toxicity of species such as O_2_^●−^ and H_2_O_2_ *in vivo* likely results from their conversion into ^●^OH radicals via the Fenton reaction [100,102]. Moreover, the binding of Fe^2+^ to DNA observed *in vivo* also promotes production of ^●^OH radicals in the vicinity of DNA, facilitating the alteration of the nucleobase and deoxyribose moieties [104]. Interestingly, several researchers have demonstrated that O_2_^●−^ also extracts iron from iron-sulfur (4Fe-4S) clusters in dehydratases present in *Escherichia coli* (*E. coli*), thus increasing cytosolic iron concentration, and facilitating increased production of ^●^OH radicals [105,106,107]. The ^●^OH radicals react with all the purine/pyrimidine bases as well as the deoxyribose backbone generating both base-derived and sugar-derived products. In addition, ^●^OH reactions with proteins surrounding DNA (e.g., histone) can produce DNA-protein cross-links.

Apart from ^●^OH radicals, several other ROS, such as ^1^O_2_ and O_3_, are also capable of reacting directly with DNA. Of the four DNA nucleobases, ^1^O_2_ oxidizes only guanine, which is the most oxidizable of the nucleobases [108,109,110]. In addition, ^1^O_2_ induces strand breaks in DNA, however, it is much less frequent than oxidation of guanine to 8-oxo-guanine [108,109,110,111]. Studies have also shown that ^1^O_2_-induced strand breaks in plasmid DNA are increased in the presence of thiols, glutathione, and cysteine, *etc.* [109]. O_3_ causes DNA damage both directly and indirectly [112,113]. Ito *et al.* [113] have shown experimentally that O_3_ reacts directly with DNA to produce the base oxidation product 8-oxo-guanine, while O_3_-induced strand breaks proceed via ^●^OH radical production.

**Table 3:** Redox potentials for some reactive oxygen species (ROS) and reactive nitrogen species (RNS) (Data taken from [101]).

Species	Reaction	*E* (V/NHE)
Hydroxyl Radical	^●^OH + H^+^ + e^−^ ⇌ H_2_O	2.80
	^●^OH + e^−^ ⇌ OH^−^	1.89
Ozone	O_3_ + 2 H^+^ + 2 e^−^ ⇌ O_2_ + H_2_O	2.08
	O_3_ + H_2_O + 2 e^−^ ⇌ O_2_ + 2 OH^−^	1.24
Hydrogen peroxide	H_2_O_2_ + 2 H^+^ + 2 e^−^ ⇌ 2 H_2_O	1.78
	H_2_O_2_ + 2 e^−^ ⇌ 2 OH^−^	0.88
Singlet Oxygen	^1^O_2_ + 4 H^+^ + 4 e^−^ ⇌ 2 H_2_O	1.79 (pH 7.0)
Carbonate Radical	CO_3_^●−^ + e^−^ ⇌ CO_3_^2−^	1.59
Dissolved Oxygen	O_2_ + 4 H^+^ + 4 e^−^ ⇌ 2 H_2_O	1.23
	O_2_ + 2 H_2_O + 4 e^−^ ⇌ 4 OH^−^	0.40
Nitrogen Dioxide Radicals	NO_2_^●^ + e^−^ ⇌ NO_2_^−^	1.04
Superoxide	O_2_ + H^+^ + e^−^ ⇌ HO_2_	−0.05
	O_2_ + e^−^ ⇌ O_2_^●−^	−0.33

Overall, the reactivity of O_2_^●−^ and ^1^O_2_ is orders of magnitude lower than that of the ^●^OH radical. The ^●^OH radical is the most reactive oxidant, with nearly diffusion-controlled rate constants. However, the half-lives and the diffusion distance of ROS, as well as the location of residues in DNA, control the efficiency of inactivation and must also be considered. For example, O_2_^●−^ has a longer half-time than the ^●^OH radical and therefore may possibly diffuse at great distances to react with DNA residues.

DNA nucleobases can also be modified by hydrated electrons (e_aq_) and H atoms which are typically produced by ionizing radiation in water; however, they are far less reactive than ^●^OH radicals [114]. While H atoms induce single strand breaks (SSBs) in DNA, they are not caused by a direct reaction with the deoxyribose backbone. Instead, the H atom reacts with a nucleobase to form a nucleobase radical, which then abstracts an H atom from the deoxyribose sugar, causing a strand break [100]. It has also been shown, both experimentally and theoretically, that hydrated electrons cannot induce strand breaks in DNA [114,115].

Similar to O_2_^●−^, nitric oxide (^●^NO) also does not react directly with DNA despite being a free radical. Instead, ^●^NO toxicity is attributed to its conversion into other RNS such as ONOO^−^, HNO_2_, and N_2_O_3_. These species are capable of modifying nucleobases and inducing DNA strand breaks via nitration and deamination. It has been observed that, at physiological pH, N_2_O_3_ is formed from ^●^NO. N_2_O_3_ directly reacts with DNA, causing nitrosation of the primary amines in DNA, which in turn lead to deamination. Specifically, N_2_O_3_ deaminates the nucleobases guanine, adenine and cytosine to xanthine, hypoxanthine and uracil, respectively. The nucleobase deamination by N_2_O_3_ causes mispairing during replication leading to mutation. Moreover, the unstable xanthine can depurinate, eventually leaving an AP site, which may then be cleaved by endonucleases to form SSBs.

While N_2_O_3_ shows reactivity to several nucleobases, ONOO^−^ reacts only with guanine. Guanine can undergo oxidation or nitrosation by ONOO^−^ to produce 8-oxo-guanine and 8-nitro-guanine, respectively. Interestingly, 8-oxo-guanine is more susceptible to oxidation by ONOO^−^ than guanine itself. Base modification by ONOO^−^ also leaves an AP site which can lead to the formation of an SSB. ONOO^−^ concentrations as low as 2 μM have been shown to cause strand breaks [116]. ONOO^−^ also directly attacks the sugar phosphate backbone of the DNA by abstracting an H atom from the deoxyribose, which then opens the deoxyribose sugar generating strand breaks.

This section describes the kinetics of the reactions with nucleobases and DNA, and summarizes well-established reactions and products that result from DNA residue modifications upon interactions with ROS/RNS that are produced either in APPs or in biological systems (e.g., culture medium, cells) treated by APPs.

### 2.1. Reactivity of ROS towards Nucleobases

The kinetics of the ROS reactions with nucleobases were determined using pulse radiolysis and laser flash techniques [93,117,118,119,120]. The oxidized products of nucleobases and DNA have been analyzed using many analytical techniques, including capillary electrophoresis (CE), thin-layer chromatography (TLC), liquid chromatography (LC), LC-mass spectrometry (LC-MS), gas chromatography-mass spectrometry (GC-MS), and immune-based detection. Descriptions and advances made in these techniques can be found elsewhere [121].

Cadet *et al.* [122] reported that a superoxide radical does not oxidize DNA. Among the nucleobases, guanine is oxidized most easily, but it has no reactivity with O_2_^●−^ [123]. However, the guanine radical, observed in several oxidative systems, can be oxidized by O_2_^●−^, yielding derivatives of guanine 5-hydroperoxides, imidazolone, and oxazolone as the oxidized products [123]. A study performed by Lafleur *et al.* [124] showed that oxidation of guanine only occurs in the reaction of ^1^O_2_ with DNA. This selective oxidation was observed to yield derivatives of 8-oxo-7,8-dihydroguanine, guanidinohydantoin, dehydroguanidinohydantoin, and spiroiminodihydantoin [92,125].

**Figure 2:**
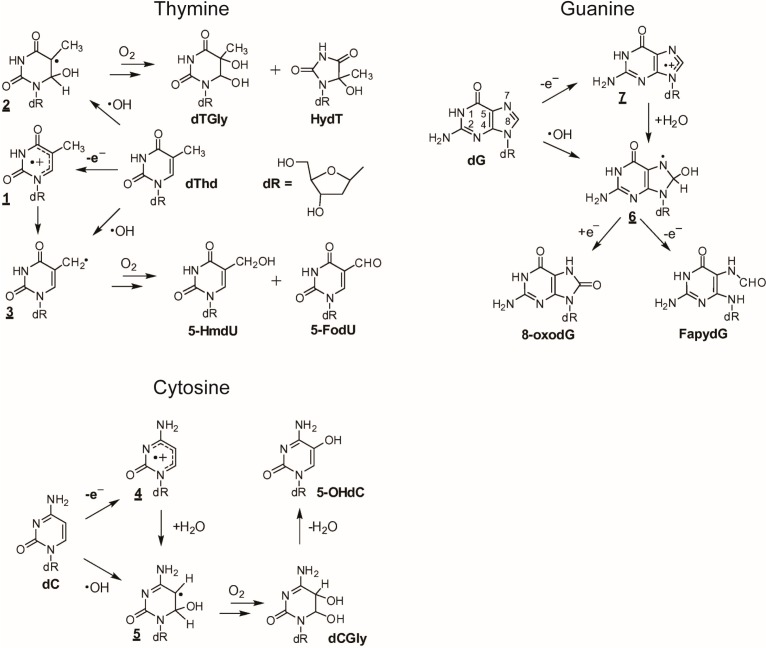
One-electron oxidation and ^●^OH-mediated oxidation of thymine, guanine and cytosine. Reproduced from [117] with the permission of Elsevier, Inc., 2014.

The rate constants for the reactions of nucleobases and DNA with ^●^OH are given in Table 4. The diffusion-controlled rate constants represent the electrophilic nature of the ^●^OH radicals. Thus, ^●^OH radicals may damage DNA, and they can attack different components of DNA indiscriminately. The ^●^OH radicals react mainly with heterocyclic bases, resulting in heterocyclic-derived radicals that are irreversibly transformed. The products of the oxidation of thymine, cytosine, and guanine by ^●^OH radicals and one-electron oxidants are presented in Figure 2 [117], which also demonstrates the basic similarities and differences between ^●^OH and a one-electron oxidant.

The initial steps of the thymine reaction with ^●^OH are addition and hydrogen abstractions to form radicals. The hydration of a thymine radical cation (**1**) produces a 6-hydroxy-5,6-dihydrothymin-5-yl radical (**2**). The formation of a 5-(uracilyl)methyl radical (**3**) occurs through ^●^OH mediated hydrogen-atom abstraction from the methyl group of thymine. The transient step of the formation of cytosine radical cations (**4**) upon one-electron oxidation is followed by the conversion to 6-hydroxy-5,6-dihydrocytosyl radicals (**5**). This radical is then converted into 5,6-dihydroxy-5,6-dihydro-2'-deoxycytidine (dCGly), which transforms to 5-OHdc through dehydration. The addition of an ^●^OH radical to the C8 position of a purine moiety is a minor pathway and results in the formation of the transient 8-hydroxy-7,8-dihydroguanyl radical (**6**). This radical can also be generated by the hydration of a purine radical cation (**7**), the one-electron oxidizing product of dG. A one-electron reduction of (**6**) forms a derivative of 2,6-diamino-4-hydroxy-5-formamidopyrimidine (FapydG). However, a one-electron oxidation of (**6**) yields 8-oxoGuo. Table 4 provides a summary of products formed from the oxidation of DNA residues.

**Table 4:** Rate constants (in L·mol^−1^·s^−1^) and major products for reactions of nucleobases and DNA with ^●^OH ([92,96,126]).

Substrate	p*K*_a_	^●^OH ^1^	Major Products of Residues
Adenine	4.15, 9.8	6.1 × 10^9^	dR-derivatives of 4,6-diamino-5-formamidopyrimidine, 8-oxo-7,8-dihydroadenine and, in some cases, 2-hydroxy-adenine also forms.
Cytosine	4.6, 12.2	6.1 × 10^9^	dR-derivatives of 5,6-dihydroxy-5,6-dihydrocytosine, 5-hydroxycytosine,6-hydroxy-5,6-dihydrocytosine, 5,6-dihydroxy-5,6-dihydro-uracil, 5-hydroxyuracil, 5-hydroxyhydantoin, trans-1-car-bamoyl-4,5-dihydroxyimidazolidin-2-one and dR-isodialuric acid.
Guanine	3.2, 9.8	9.2 × 10^9^	dR-derivatives of imidazolone, oxazolone, 8-oxo-7,8-dihydroguanine and 2,6-di-amino-4-hydroxy-5-formamidopyrimidine. In addition, 1,N(2)-adducts of glyoxal and propene to guanine can be formed.
Thymine	9.9, >13	6.4 × 10^9^	dR-derivatives of 5,6-dihydroxy-5,6-dihydrocytosine, 5-hydroxycytosine, 6-hydroxy-5,6-dihydrocytosine, 5,6-dihydroxy-5,6-dihydro-uracil, 5-hydroxyuracil, 5-hydroxyhydantoin, trans-1-car-bamoyl-4,5-dihydroxyimidazolidin-2-one and dR-isodialuric acid.
DNA	-	4.0 × 10^8^	

^1^ at pH 7.0.

### 2.2. Reactivity of RNS towards Nucleobases

**Figure 3:**
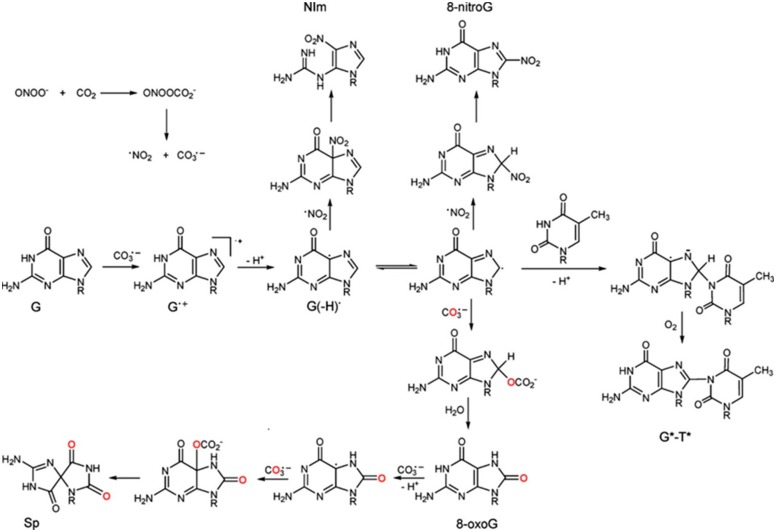
Lesion derived from the oxidation of guanine in DNA by decomposition products of nitrosoperoxycarbonate (CO_3_^●−^ and ^●^NO_2_). Adapted from [127] with the permission of the American Chemical Society, 2011.

The redox potentials given in Table 2 indicate that there is no reaction of ^●^NO_2_ with guanine. However, the major oxidized product of guanine (e.g., 7,8-dihydro-8-oxyguanine (8-oxo-G)), which has a redox potential of 0.74 V *vs.* NHE at pH 7.0, can react readily with ^●^NO_2_ (*k* = 5.3 × 10^6^ M^−1^·s^−1^). The products of the nitration reaction are presented in Figure 3 [127]. It appears that a combination reaction takes place either through the C5 or C8 position of 8-oxo-G. In the case of ^●^NO_2_ oxidation, both N and O atoms are delocalized and therefore can be involved in the formation of chemical bonds with the target molecule, guanine. The oxidized products indicate N–C bond formation. Formation of 8-nitroguanine (8-nitroG) occurs by the addition of ^●^NO_2_ to the C8 position. The attack of ^●^NO_2_ at the C5 position generates unstable adducts, which rapidly decompose to 5-guanidino-4-nitroimidazole (NIm), as presented in Figure 3.

**Figure 4:**
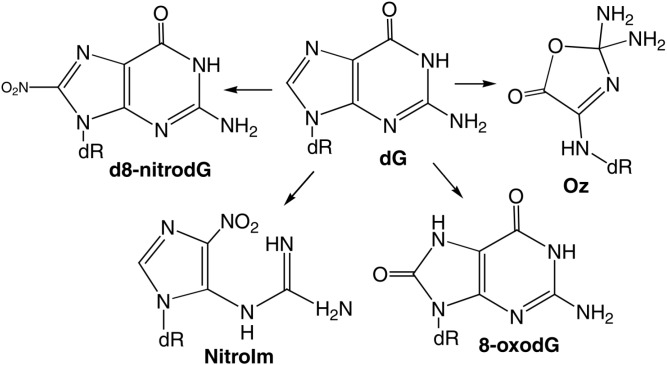
Products of peroxynitrite oxidation of dG. Adapted from [94] with the permission of Elsevier, Inc., 2004.

The main oxidized products from reactions of guanine with ONOO^−^ and ONOOCO_2_^−^ include 8-oxy-2'-deoxy-guanosine (8-oxo-dG) and 8-nitro-2'-deoxyguanosine (8-nitro-dG), as displayed in Figure 4 [94].

**Figure 5:**
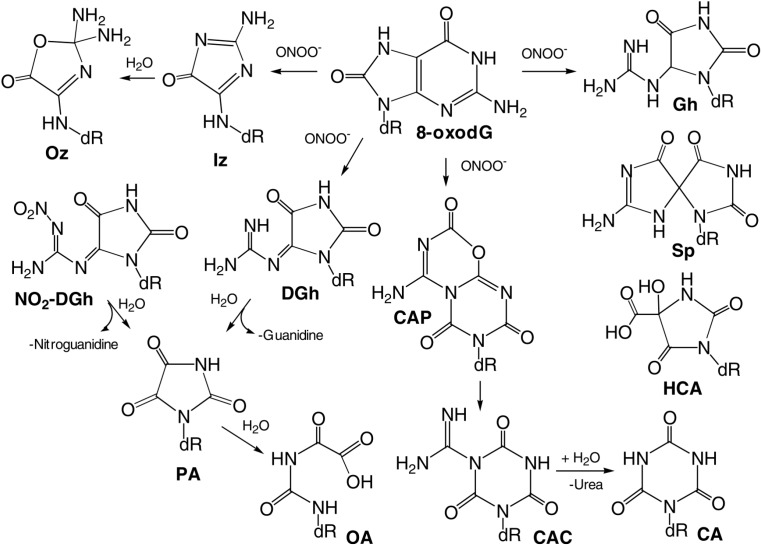
Products of peroxynitrite oxidation of 8-oxodG. Adapted from [94] with the permission of Elsevier, Inc., 2004.

Both of these products depurinate rapidly to give off 8-nitro-G, 5-guanidino-4-nitroimidazole (NitroIm) and 2,2-diamino-4-[(2-deoxy-β-d-*erythro*-pentofuranosyl)amino]-5(2H)-oxazolone (oxazolone (Oz)). The latter is the stable product of 2-aminoimidazolone (Iz). Significantly, 8-oxo-G is much more reactive than the parent guanine and therefore, several secondary oxidized products, such as spiroiminodihydanton (Sp) and guanidinohydantoin (Gh) are also obtained (Figure 5) [94].

The distribution of products depends on the concentrations and fluxes of oxidants. At low concentrations and fluxes, Sp and Gh are produced predominantly, while 2,4,6-trioxo [1,3,5] triazinane-1-carboxamidine (CAC), dehydroguanidinohydantoin (DGh) and NO_2_-DGh are the major products at high fluxes [128]. Comparatively, oxidation of guanine and 8-oxo-G by CO_3_^●−^ produces Sp as the major product. This is demonstrated in the oxidation of guanine in DNA by CO_3_^●−^ and ^●^NO_2_, which are decomposed species of nitrosoperoxycarbonate (Figure 3) [93,127,129]. The reactions of CO_3_^●−^ with guanine in 2'deoxyoligoribonucleotides produce a cross-linked guanine-thymine product (G*-T*) with a covalent bond between C8 (G*) and thymine N3 (T*) atoms (Figure 3). This cross-linked product is also produced in the reaction of native DNA with 0.1 mM peroxynitrite in the presence of a 25 mM bicarbonate/carbonate solution at pH 7.5–7.7 [127,130].

### 2.3. DNA Strand Breaks Induced by ROS/RNS

**Figure 6:**
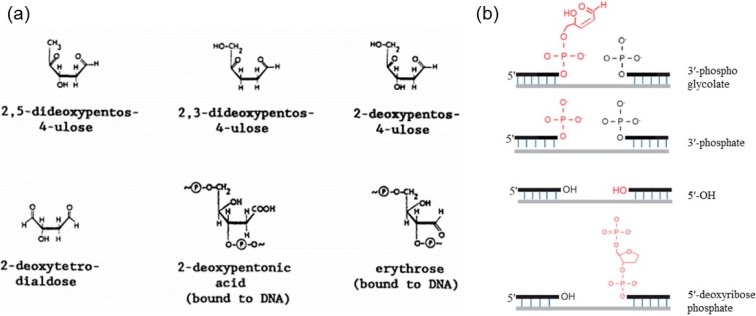
ROS/RNS induce strand breaks in DNA: (**a**) ^●^OH radical-induced products of the deoxyribose sugar in DNA (adapted from [102] with permission from Elsevier, Inc., 1991); and (**b**) ROS-induced SSBs containing blocked termini such as 3′-phosphoglycolate, 3′-phosphate, 5′-OH and 5′-deoxyribosephosphate (adapted from [131], 2014).

Strand breaks can occur either directly by oxidation of the deoxyribose sugar by ROS/RNS (sugar damage) or indirectly by enzymatic cleavage of the phosphodiester backbone during repair of the oxidized bases via base excision repair (BER) or nucleotide excision repair (NER) processes (repair processes detailed in Section 4.1.3). In general, base modifications induced by ROS/RNS do not produce altered sugars or strand breaks unless the altered nucleobase labilize the *N*-glycosidic bond to form an AP site which is then removed by β-elimination. The damage to the sugar moiety occurs typically due to hydrogen abstraction from the deoxyribose. The H atom abstraction from the C4' position of deoxyribose generates a deoxyribose radical [100], which in turn reacts further causing the release of intact nucleobases, alteration of other deoxyribose moieties, and eventually strand breaks in the DNA. While some of the altered deoxyribose is released from the DNA backbone, some remains in the backbone, forming “alkali-labile” sites. Some of the typical products of ^●^OH radical interaction with deoxyribose in DNA identified using the GC/MS technique are shown in Figure 6a. The ^●^OH-induced sugar products include 2,5-dideoxypentos-4-ulose, 2,3-dideoxypentos-4-ulose, 2-deoxypentos-4-ulose, 2-deoxytetrodialdose, 2-deoxypentonic acid and erythrose. The SSBs induced by ROS/RNS have blocked termini such as 3'-phosphoglycolate, 3'-phosphate, 5'-OH and 5'-deoxyribosephosphate, as shown in Figure 6b [131].

During the repair of nucleobases altered by ROS/RNS via the BER and NER processes, the excision of two altered nucleobases located close to each other on opposite strands can cause a double strand break (DSB) in the DNA [43,132,133]. Moreover, SSBs generated by ROS/RNS can also be converted into DSBs during normal replication of the DNA [134,135,136].

In addition to specifically attacking the DNA, ROS/RNS can also attack DNA indirectly through reaction products generated via their interaction with other biomolecules such as lipids and proteins [91,103,137]. The end-products of lipid oxidation by reactive species, such as malondialdehyde, can bind to DNA to induce mutations. Moreover, ROS/RNS may also directly damage DNA damage repair enzymes and polymerases, thus slowing the repair processes or preventing replication altogether [91,103,137].

Another factor affecting the stability of DNA structure is pH [138]. A pH of less than 4 (mildly acidic) results in the hydrolysis of the *N*-glycosidic bond, thus separating nucleobases from the deoxyribose backbone. A pH of less than 1 (very acidic) leads to hydrolysis of both the *N*-glycosidic bond and the phosphodiester bond separating nucleobases, deoxyribose and phosphates [138]. In comparison, a pH of more than 11.3 (basic) alters the polarity of hydrogen-bonded groups and causes the separation of the two complementary strands, leading to DNA denaturation [138].

In summary, nucleobases are susceptible to damage by ROS and RNS. Modifications of the nucleobases alter the specificity of their hydrogen bonding. As a consequence, nucleobase oxidation and deamination products, if left unrepaired, can cause base mispairing (G→T transversion and G:C→A:T transitions) during replication, thereby causing mutations. In mammalian cells, a complex signaling pathway called “DNA Damage Response” (DDR) is activated in response to DNA damage and ultimately decides the fate of a cell-cell cycle arrest and DNA repair, cell death, or mutation (detailed in Section 4.1). While DNA repair systems exist in biological systems for the successful removal of modified bases, failure to repair these irregular bases can have serious biological consequences. DNA glycosylases are a family of enzymes that initiate repair processes by hydrolyzing the *N*-glycosidic bond and thereby isolating the modified base from the deoxyribose moiety of the DNA. DNA glycosylases are involved in the repair of both oxidized and deaminated bases. Removal of the modified base creates an AP site, which is then processed by AP endonucleases that cleave the phosphodiester bond at the AP site and create a nick in the strand. Typically, DNA polymerase β then adds a single nucleotide, and DNA ligase seals the nick. However, failure to do so will leave a break in the strand, thus creating SSBs and DSBs.

## 3. APP Interactions with Isolated DNA

A comprehensive understanding of the physical and chemical processes governing DNA damage under various APP conditions is crucial for the development of biomedical applications using plasma. The control of DNA damage initiated by plasma treatment can be beneficial in some applications (e.g., cancer therapy); however, for other applications (e.g., wound healing), it is necessary to avoid DNA damage. Therefore, in order to elucidate plasma-mediated DNA-alteration, as well as DNA protection mechanisms against plasma, it is necessary to investigate the effects of APP on DNA that is isolated or surrounded by compounds that can be found in the vicinity of the DNA in a cell. This section primarily describes the experimental efforts of a number of research groups involved in investigating plasma exposure conditions that govern DNA strand break formation. These groups have also made an attempt to evaluate the physical and chemical factors in plasma that are responsible for alterations in DNA.

### 3.1. Types of DNA Damage Induced by APPs in Isolated DNA

In order to estimate the effect of APPs on isolated DNA molecules, the two most common techniques used are agarose gel electrophoresis [12,29,45,47,48,49,50,51,52,53,54,55,56,57,58] and molecular combing [59,60,61].

**Figure 7:**
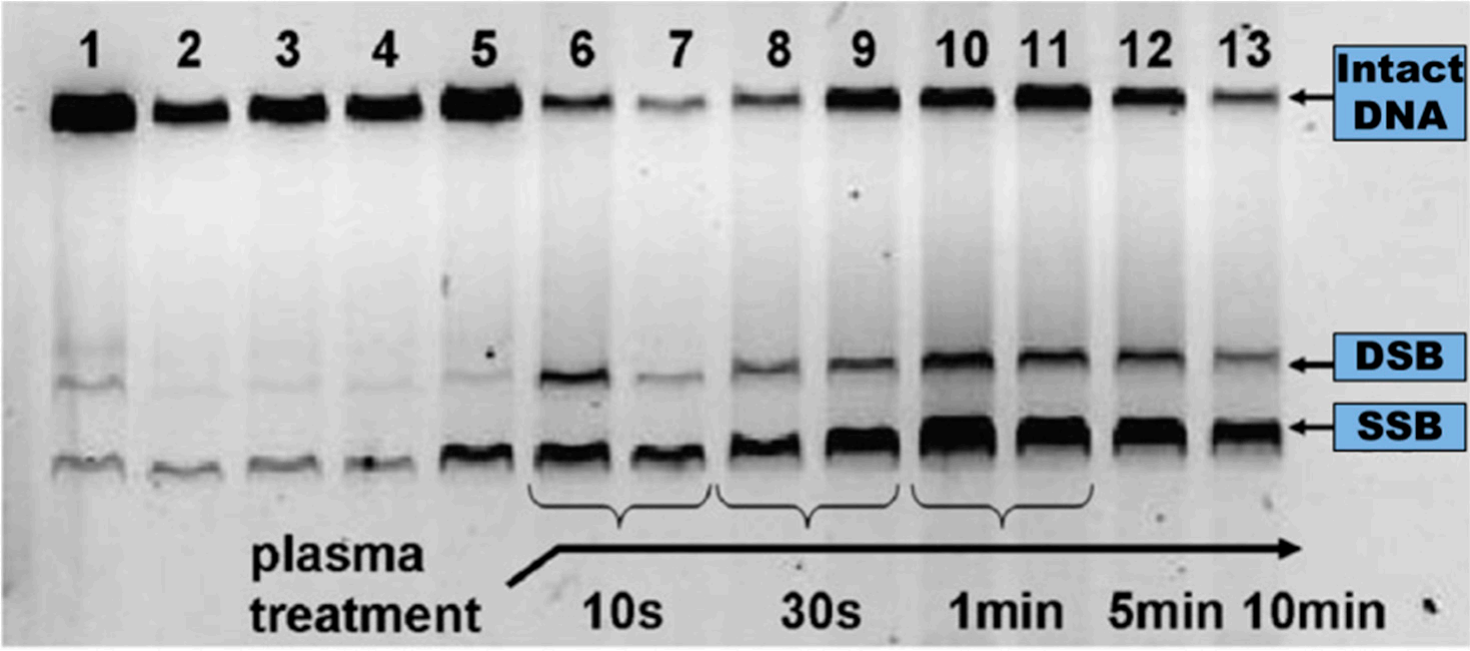
Typical image of agarose gel displaying thirteen samples of dry plasmid DNA treated under different conditions: (1) DNA in aqueous solution; (2) and (3) DNA placed on a mica substrate; (4) and (5) DNA placed on mica and treated with He gas; from (6) to (13) DNA placed on mica and treated by the APP jet at various exposure times. Adapted from [53], 2010.

Agarose gel electrophoresis has been used for the assessment of damage in different types of plasmid DNA treated by APPs (e.g., pBR322 [45,47,53,54], pUC18 [12,57,58], pAHC25 [55], pCDNA3.1 [52], and hrGFP-II-I [29]). This technique can be used to separate DNA fragments with respect to their different lengths or the topological conformations that result from strand break formation [139]. A typical agarose gel image taken by a UV imager is presented in Figure 7. The fastest, middle, and slowest bands represent the supercoiled conformer (indicating undamaged plasmid DNA), the linearized conformer that forms due to a single event of DSB formation, and the open circular conformer that results from SSBs, respectively. The fluorescent intensity of these bands represents the amount of the corresponding conformers in DNA samples, which were treated under specific conditions and then stained with SYBR Green or ethidium bromide dyes.

**Figure 8:**
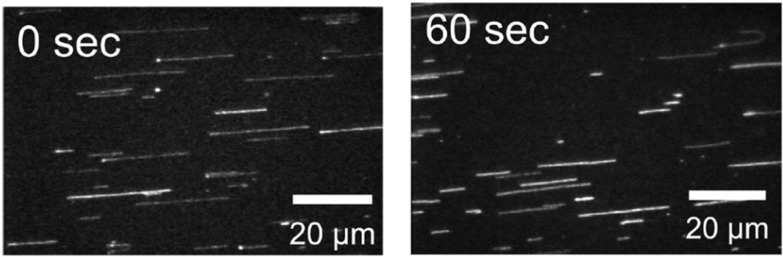
Typical photographs of DNA molecules after combing: before (0 s) and after APP treatment of 1 min. Adapted from [60] with the permission of AIP Publishing LLC, 2011.

Molecular combing, which is used for single molecule observations, measures the length of individual linear DNA molecules (e.g., λDNA [59,60,61]). In this technique, fluorescently stained DNA molecules from solution are adsorbed and combed on a glass coverslip. The coverslip is then dried and the sample is observed under a fluorescence microscope. The DNA length measured shows significant changes after plasma exposure by comparison to non-irradiated samples, as shown in Figure 8. The rate of strand breakage can be determined using a simple mathematical model from the measurement of relative changes in the length of the DNA as a function of plasma exposure [61].

Some of the other techniques used include polymerase chain reaction (PCR) [55], Fourier transform infrared (FTIR) spectroscopy [58], Raman spectroscopy [57], matrix-assisted laser desorption ionization-time of flight mass spectrometry (MALDI-TOF) [50], and high-performance liquid chromatography-tandem mass spectrometry (HPLC-EIS-MS/MS) method [62]. Most of these methods are used for the detection of DNA constituents, such as oligonucleotides (single- [57,58] and double-stranded [57]) and 2-deoxyguanosine [62].

A number of research groups have focused on the formation of strand breaks in plasmid DNA that are caused by plasma exposure and lead to alterations in topology. In order to eliminate contributions from the medium in which DNA is placed during plasma treatment, plasmid DNA was dried and exposed directly to APPs [49,53,57,58].

Ptasinska *et al.* [53] and Kim *et al.* [49] observed rapid degradation of supercoiled DNA within the first few seconds of plasma treatment for APPs ignited both in inert gas (*i.e.*, He) [53] and in the He/O_2_ mixture [49]. The increase in DNA damage reached ~80% after 10 min of He APP irradiation. This 10-min APP treatment yielded 70% production of SSBs and 10% production of DSBs. A more dramatic damaging effect on dry plasmid DNA was observed when the APP was used with an oxygen admixture [49]. Exposure for longer than 20 s resulted in complete DNA degradation due to the production of multiple fragments. Most of the studies on strand break formations in DNA were performed in an aqueous DNA solution [12,29,47,48,52,54,55,61], and all of the studies showed that water failed to protect the plasmid from APP species, even under different experimental conditions.

Lackmann *et al.* [57,58] performed a series of experiments in which different plasma components (*i.e.*, vacuum UV (VUV) and reactive particle components) were separated. These components were used for DNA plasmid treatment in a He environment. The authors detected SSB and DSB formations in plasmid DNA exposed to particle components of the APP, while SSB and dimers formed due to the VUV component [58]. Moreover, they transformed plasma-treated DNA into *E. coli* cells that resulted in reduction of transformation efficiencies by comparison to untreated DNA, most likely due to mutagenic effects [57].

As for results with dry DNA, Yan *et al.* [55] reported that the abundance of the supercoiled form of plasmid DNA in aqueous solution decreased, while that of the open circular and linearized forms of plasmid DNA increased with increased treatment times. The authors observed approximately 90%, 40% and no supercoiled DNA conformers at plasma treatment times of 1, 2, and 4 min, respectively. Further increases in plasma exposure exhibited a gradual degradation of linear DNA and formation of smaller DNA fragments, which were detected as smeared bands on the agarose gel image [50,55]. Leduc *et al.* [29] observed that, under their experimental conditions, APP exposure for 30 s was sufficient to degrade the plasmid completely.

In the studies mentioned above, the distance between the plasma source and the DNA sample was fixed; however, it is known that the distribution of reactive species varies depending on the location within and around the APP jets [140]. Bahnev *et al.* [47] measured the radial and axial lengths of the visible zone of the plasma jet to be 0.4 and 5.5 cm, respectively, whereas plasmid DNA damage was detected at distances of 2 cm radially and 25 cm axially from the source. The highest damage to DNA detected (~60%) was at the tip of the plasma jet, followed by a gradual decrease in the axial direction. In contrast, using another APP source [12], the level of damage was shown to remain constant (90%–80%) along the entire length of the visible zone of the jet, after which it dropped dramatically outside the zone. These discrepancies can be explained by different experimental parameters of the APP sources used, such as the input power, DC pulses *vs.* AC pulses, and so on. For example, the contribution to plasmid DNA damage was studied by varying both the distance from the plasma source and the exposure time for two different electrical parameter settings with respect to the power of the plasma source [12]. The trends in DNA damage observed for both spatial and temporal factors were comparable, but showed a difference in the relative yield of damage. Moreover, the formation of DSBs was observed only in the higher power plasma source condition. Li *et al.* [50] performed similar studies in which the genetic effects of the plasma jet became more significant with an increase in the source power, with other parameters held constant. In the lower power range (10–20 W), the authors primarily observed the formation of SSBs, while above 60 W there was a significant yield of DSBs. A further increase in power led to the total degradation of plasmid DNA. In addition, varying other parameters of the plasma source (e.g., gas flow rate affects fluxes of chemically active species and induces evaporation of an irradiated sample, thus affecting the volume and concentration of a DNA solution) can influence the degree of DNA damage [50].

**Figure 9:**
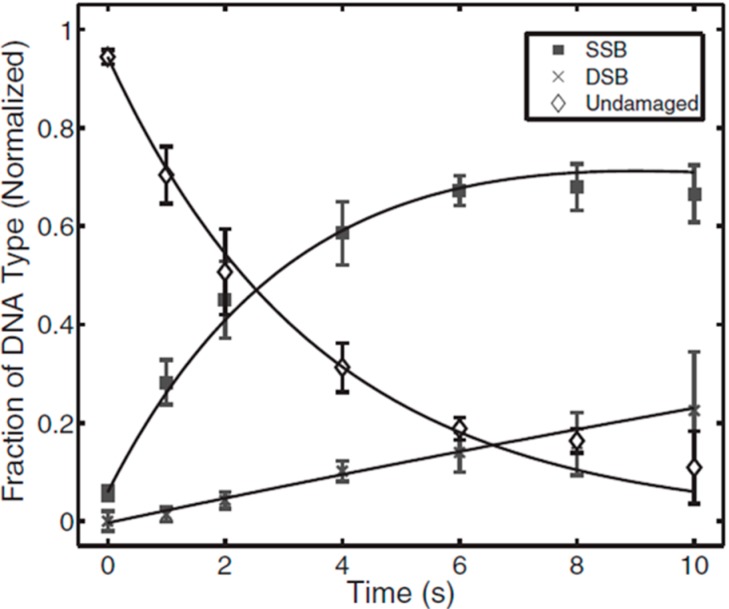
Representation of the relative abundances of three DNA conformers: supercoiled form-undamaged DNA, open circular form-SSB, and linear form-DSB in PBS solution treated by APP at various exposure times. Adapted from [52] with the permission of AIP Publishing LLC, 2011.

In order to study APP effects on the formation of strand breaks in DNA under more realistic conditions, plasmid DNA was resuspended in the following buffers: PBS (phosphate buffered saline, which contains sodium chloride and sodium phosphate) [29,45,51,52], and TE (tris-EDTA) [52,56]. Two water and PBS comparison studies of plasmid DNA degradation reported contradictory results [29,52], which may be due to different buffer concentrations. Leduc *et al.* [29] suggested that the buffer is partially responsible for plasmid DNA protection, because PBS reduced plasmid DNA damage compared to DNA in aqueous solution. However, an experiment performed by O’Connell *et al.* [52] resulted in a yield of DNA damage that was quantitatively similar but showed a different rate for strand break formation in plasma-irradiated DNA in aqueous and PBS solutions. The time scale for total degradation of supercoiled DNA was approximately one order of magnitude longer in PBS than in water. The rate of SSB and DSB formation for DNA in PBS is presented in Figure 9.

As seen for dry plasmid DNA and for plasmid DNA resuspended in aqueous solution, Alkawareek *et al.* [45] reported rapid damage upon plasma exposure, with complete loss of the supercoiled DNA conformation after 90 s. The formation of DSBs occurred as early as 10 s and reached ~35% at 60 s. While PBS is a moderate radical scavenger, TE is known to be a strong radical scavenger, in particular of OH radicals [141]. Therefore, DNA in TE buffer can be used to evaluate damage to DNA induced by plasma radicals and/or can mimic the radical scavenging environment found in cells in order to protect DNA from damage. As reported by O’Connell *et al.* [52], DNA damage upon plasma exposure is reduced significantly compared to damage in aqueous or PBS solutions. In their investigations, there was clear evidence of DNA damage in water, whereas there was only minor formation of strand breaks in TE solution. These studies definitely indicate the importance of radicals in DNA damage [52]. However, in an experiment performed by Kim *et al.* [56], an enhancement of strand break yields for DNA in TE buffer was observed by adding oxygen to the flow of the inert gas to increase oxygen reactive species.

To approach realistic conditions even more closely, a study of the influence of amino acids on DNA strand break formation was performed by Stypczynska *et al.* [54]. Plasma irradiations were conducted for different molar ratios and for two different amino acids, glycine and arginine, and the authors observed a decrease in the strand break yields for both amino acids. In order to quench the occurrence of DSBs, the addition of a small amount of amino acid was sufficient (e.g., an amino acid to nucleotide ratio of 0.5:1), while the yield of SSBs remained the same up to an amino acid to nucleotide ratio of approximately 4:1. The authors concluded that the changes in the yield of strand breaks due to the presence of amino acids were determined not only by the physical shielding of DNA, but also by the interactions of radicals formed from amino acids upon plasma irradiation. These two competing processes, protection and damage due to plasma-induced radicals, can occur in the cell and depend on the type of compounds that surround the DNA. It is worth noting that other bio-macromolecules (e.g., proteinase K) were also altered during plasma irradiation; however, the rate of inactivation was significantly lower than the damage rate of plasmid DNA under the same experimental conditions [45]. This was explained by the fact that, in order to inactivate an enzyme, many different physicochemical events must be accumulated, whereas plasmid DNA can be damaged by just a single DSB event [45]. The authors concluded that DNA might be a more sensitive cellular target than some enzymes.

Leduc *et al.* [29] treated plasmid DNA in a complex culture medium that consisted of a carbonate buffer with salts, amino acids, a phenol indicator and vitamins required for cell growth. The results were compared to those for DNA in aqueous and PBS solutions irradiated under the same plasma experimental conditions. They observed that even after maximum operational plasma exposure, the DNA in the medium remained unaffected. The authors suggested that some components in the medium were able to protect the plasmid DNA from plasma degradation, but the effect of the composition of the medium on plasma degradation was not explained. The same group obtained similar results using another plasma source to assess the possible effects of direct and indirect plasma treatment on isolated plasmid DNA in these three different environments [46]. DNA was unaffected when plasma treatment was carried out in the culture medium, whereas the plasmid was destroyed completely after 30 and 60 s of plasma treatment in aqueous and PBS solutions, respectively. In comparing results from the two different plasma sources, the authors reported that direct plasma treatment is more severe than indirect treatment, which confirmed previous studies [142].

**Figure 10:**
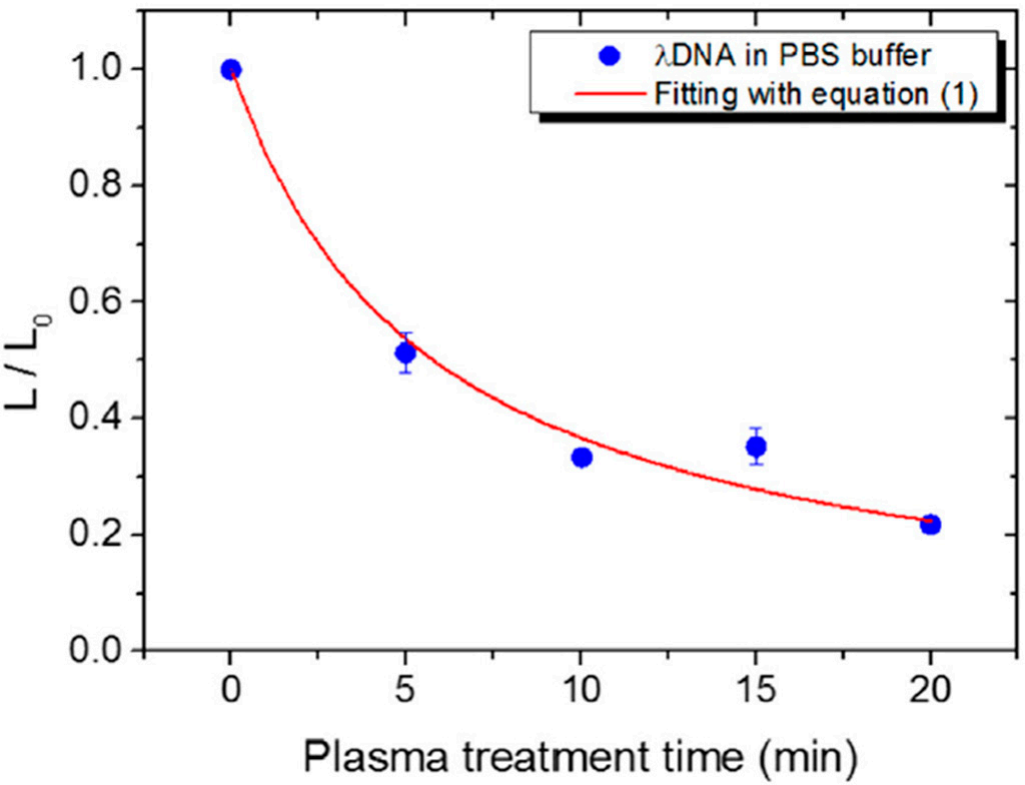
Representation of the rate of change of DNA molecule average length (where L_0_ is the DNA length before plasma exposure, and L is the length after the exposure) in PBS solution treated by APP at various exposure times. Adapted from [59] with the permission of Elsevier, Inc., 2014.

Significant changes in the length of isolated linear DNA molecules were also recorded [59,60,61]. Studies of plasma-irradiated DNA in an aqueous solution resulted in a number of fragmented, short DNA molecules, which indicates that the relative DNA length decreased exponentially with increased exposure time [60], as shown in Figure 10, and as observed for strand break formation detected by the gel electrophoresis technique [12,29,47,48,52,54,55,61]. Moreover, the DNA DSB cutting rate for DNA fragmentation increased proportionally to the discharge power [60].

In an experiment by Antoniu *et al.* [59], DNA in solutions with different pH was irradiated by APP, and DNA fragmentation was correlated to cell viability under the same plasma treatment conditions. In the case of citric acid (pH 4), DNA samples were exposed to plasma for up to 2 min, while PBS (physiological pH) required longer treatment times, most likely because of the nontoxicity of PBS and its ability to maintain the pH and prevent the denaturing of cellular DNA [59]. The reduction in the average relative length of DNA, in both citric acid and PBS buffers, was significant after exposure to APP. The molecule length decreased by ~40% after 2 min, yielding a DNA DSB cutting rate of 0.17/min and 0.21/min in PBS and citric acid buffers, respectively. After 20 min of APP treatment, the average relative length reached only 20% of the normalized length value of DNA in PBS [59]. The authors reported that DNA experienced an average of 2.5 DSBs/molecule and the *E. coli* decontamination value was 14.5 min when treated in the PBS buffer, whereas 0.29 DSBs/molecule and a decontamination value of 1.4 min were obtained when treated in the citric acid buffer [59]. These results suggest that the citric acid medium is approximately ten times safer for DNA and more effective for sterilization [59]. Further, Kurita *et al.* [61] evaluated the protective effect on DNA of antioxidant agents such as ascorbic acid, glucose, and sodium azide. The relative DNA length decreased gradually with increased exposure time for all three agents [61]. However, in the cases of ascorbic acid and glucose, this decrease was suppressed with increasing concentrations of the antioxidant reagents. The authors reported that even several tens of micromoles of these two agents were sufficient to prevent a length reduction in half of the DNA molecules. Their experiment also showed that glucose exhibited a higher protection potential than did ascorbic acid. For sodium azide, which is a specific scavenger of ^1^O_2_, no significant protective effect was detected. In addition, the authors reported that the pH of water remained above 6 following APP treatment; hence, the influence of pH on DNA damage can be considered negligible [61].

It is interesting to note that even when protection of a plasmid DNA against plasma treatment was observed, DNA damage was detected in more complex environments, (*i.e.*, in the cell). This point will be discussed further in the next section.

Other techniques (e.g., MALDI-TOF, HPLC) have shown strong plasma-induced fragmentation of DNA analogues, such as oligonucleotides [50] or the production of oxidized nucleoside from 2-deoxyguanosine [62]. In a comparison of mass spectra of treated *vs.* untreated oligonucleotide samples, Li *et al.* [50] found additional evidence for the formation of small fragments induced by APP. Sousa *et al.* [62] reported that oxidized nucleoside production increased nearly linearly with exposure to ^1^O_2_ formed by atmospheric pressure microdischarges. The authors showed that ^1^O_2_ induced the formation of hydroxy-8-oxo-4,8-dihydro-2-deoxyguanosine followed by nucleoside oxidation. Further, other oxidized nucleoside products formed by secondary decomposition of transient oxidized species were observed [62]. They also observed a decrease in pH from 6.8 and 5.5 before treatment to a pH of 4 after only 2 min APP treatment when two different sources of water was used, while no change in pH change was observed in a buffered aqueous solution. Hence, they concluded that the pH of the treatment solution influences the type and amount of APP-induced DNA damage [63]. Lackmann *et al.* studied oligonucleotides with 18-nucleobabses of T (dT_18_) [58], C (dC_18_) and G (dG_18_) [57]. A comparison of FTIR spectra for single-stranded dT_18_ before and after treatment with the VUV component showed loss of C=C bonds and formation of C-C bonds indicating to thymine dimer formation [58]. Raman spectra of single-stranded dG_18_ treated by APP indicated breakage or modification of DNA strands and nucleobase alteration, while spectra of double-stranded dG_18_: dC_18_ showed only minor changes [57]. These results have proven that single-stranded DNA has a higher sensitivity to APP treatment than does double-stranded DNA [57].

By using the PCR technique for amplification of specific segments of DNA, Yan *et al.* [55] conducted experiments with three types of genes that were exposed to APP treatment. The authors concluded that, under proper conditions, APP exposure does not affect the genes of plasmid DNA.

### 3.2. Evaluation of Effects of APP Components on Strand Break Formation

As presented above, the effects of APP on isolated DNA in different environments have been studied extensively; however, a question remains: What is the mechanism of strand break formation? In order to approach this issue, many groups have explored which plasma components are the most efficient in producing DNA damage. A recent review summarized results from computational simulations of plasma-biomolecule and plasma-tissue interactions in which two types of operative factors, chemical and physical, were considered [143]. Chemical factors included radicals, ions, and neutral molecules lead to chemical reactions, while physical factors included heat, electric fields, UV radiation and surface charging.

Here, our primary focus is on the findings that deal with these two operative factors from an experimental point of view. There is a strong consensus among many research groups that the most likely factor that causes strand breaks in DNA comes from the chemically active species. The technique most commonly used to determine reactive species in APP is optical emission spectroscopy. Two parameters influence gas composition in APP, input power and the type of discharge gas [59]. Most of the studies of APP effects on DNA were performed using a pure inert gas (e.g., He [12,47,50,53,54,59] or Ar [60,61]), or an inert gas with O, NO, or an air admixture (e.g., He/O_2_ [45,49,51,52,55,56,57,58], Ar/air [48] and He/O_2_/NO [62]). The most dominant emission bands observed in optical spectra of an APP zone corresponded to excited chemically nonreactive N_2_, N_2_^+^, and He species. Relatively lower-intensity emission bands were observed for chemically reactive oxygen and nitrogen species, such as O, O_3_, ^1^O_2_, ^●^OH, and ^●^NO. All of these reactive species are known to be very destructive to biomolecules and are likely involved in synergistic processes that lead to DNA damage [52]. In order to further increase the reactivity of APP, molecular gases were added to the inert gas flow. However, it has been observed that only a limited amount of oxygen can be introduced to the inert gas flow in which APP can be sustained [56]. This drawback prevents a higher production of reactive species in the APP zone. However, despite the relatively low concentration of ^●^OH or other ROS, the concentration of these species increased significantly due to collisions between plasma components and molecules in the surrounding air [47,48].

O’Connell *et al.* [52] and Niemi *et al.* [51] correlated the formation of DSBs with atomic oxygen density. The authors measured rates for SSB and DSB formation as a function of absolute atomic oxygen density formed in the core of the plasma bulk. However, they reported that the assumption that the atomic oxygen itself is responsible for DSB production has not been confirmed. They also stated that the density of other neutral components in the APP jet can be effective in inducing DNA damage. In contrast, the rate of SSB formation showed no evidence of any correlation with atomic oxygen density. The possibility of DNA damage by radicals was also suggested by Leduc *et al.* [29] from their experiment in which plasmid DNA was treated under the same conditions, but with DNA resuspended in water, PBS, and culture media. There was no DNA damage observed in the media, which contained radical scavengers, such as vitamins (present in the media, but not in the PBS). Therefore, the protective effect of the media was attributed to the presence of components that scavenge radicals and to the presence of charged plasma species, as well as to the different buffering capability of the media [29]. These findings were confirmed by Kurita *et al.* [61], who found that DNA fragmentation was reduced significantly due to protection from antioxidant agents during APP exposure. Sousa *et al.* [62] also stressed the importance of oxygen radicals on nucleoside modifications; however, other reactive species that can be byproducts of NO (*i.e.*, NO_2_, NO_3_, N_2_O_5_, and HNO_3_) should not be ruled out, particularly in the formation of decomposition products. Ptasinska *et al.* [53] estimated that ~60% of the total damage to plasmid DNA is caused by excited and reactive species.

Regarding physical operative factors, Li *et al.* [50] concluded that no thermal factor contributes to DNA damage, because the APP jet used in their experiment had a very low temperature. Moreover, no intense electric field was detected, therefore this was also excluded as a contributor to strand break formation. Similar findings were obtained from molecular dynamics simulations, which demonstrated that, although the field applied effectively created electroporation of the cell membrane, the internal structure of DNA was largely unaffected [143]. Ptasinska *et al.* [53] likewise concluded that 10% of the DNA damage observed was due to UV light that induced SSBs; however, no DSBs were detected. Li *et al.* [50] also reported a small effect of UV radiation on DNA damage. Additionally, by using electric probe measurements, they showed that the concentration of charged particles in the APP zone is relatively low and that, therefore, these particles do not contribute to DNA damage. In contrast, Ptasinska *et al.* [53] estimated that ~30% of the total DNA damage was due to the charged particles passing through a high-transmission metallic mesh with a corresponding applied voltage and polarity. However, using an electric probe or metallic mesh can perturb the electric field, and therefore, induce different plasma conditions than would be present without a probe or mesh. In another approach taken by Lackmann *et al.* [57,58], the VUV or reactive particle (primarily O_3_ and O) component was isolated and the resultant effects were compared to the total effect of APP. The VUV radiation induced SSBs, dimerization of DNA, and chemical modifications of nucleobases in single-stranded oligonucleotides. In contrast, the reactive particle component led to negligible changes in nucleobases, but induced both SSB and DSB formation.

These approaches were tested in order to find the most effective plasma component involved in DNA damage. However, the synergistic effect of many plasma components may play the most significant role in the mechanism of DNA strand breaks. Indeed, as was reported by Lackmann *et al.* [57], the effects observed for DNA treated with APP containing all components indicated much more significant changes than the sum of effects from particular APP components, thus proving the synergy of plasma components.

Formation of strand breaks induced by APPs has been studied extensively, but other changes to DNA, such as base modification, base release, oxidative DNA damage, and DNA-protein cross-links still need to be investigated further. Therefore, detection of these DNA alterations continues to be encouraged, because the outcome of such investigations will give a more comprehensive picture of DNA damage by APPs.

## 4. APP Interactions with Cellular DNA

APPs produce a variety of ROS and RNS including ^●^OH, H_2_O_2_, ^1^O_2_, O_2_^●−^, ^●^NO, ONOO^−^, *etc.*, species that can also be generated by eukaryotic cells via normal cellular metabolism. During plasma treatment, cells or tissues are exposed to numerous ROS/RNS produced directly by the plasma as well as those produced through interaction of the APPs with the surrounding medium. Inadequate neutralization of these ROS/RNS by the cellular antioxidant defense system may lead to oxidative stress, which subsequently, may induce many cytoplasmic and nuclear responses, including DNA damage, cell cycle modification and apoptosis.

APP treatment of dry or aqueous isolated DNA, detailed in the previous section, offered a simple approach to understanding the effects of various plasma species in inducing DNA damage and provided information primarily about the different types of damage to DNA. However, it is imperative to study the plasma induced DNA modifications in the context of living cells, as some of the damaging or protective effects observed in the case of isolated DNA may either be enhanced or quenched by the complex interplay between DNA damage sensing and repair mechanisms in the cell. Eukaryotic cells have a well-developed DNA damage repair system; hence, certain types of plasma-induced lesions observed in isolated DNA might not even be visible in cellular DNA. On the other hand, if not repaired properly, certain types of DNA damage are converted to a different type of DNA lesion. For example, Vilenchik *et al.* [144] estimated that during each cell cycle in a eukaryotic cell, ~1% of the SSBs are converted to ~50 DSBs. Taking this into consideration, it may be assumed that even if plasma treatment induces only SSBs, during the course of DNA damage repair, some of those may be converted to DSBs. Hence, in addition to exploring APP-induced effects on isolated DNA, plasma researchers are also investigating APP effects on cellular DNA. In this section, we briefly describe the cellular responses associated with DNA damage in eukaryotic cells, and the various repair mechanisms activated in response to oxidative DNA damage at various phases of the cell cycle. The current state of the knowledge regarding APP effects on eukaryotic and prokaryotic cellular DNA is also outlined.

### 4.1. Cellular Responses to DNA Damage in Eukaryotic (Mammalian) Systems

#### 4.1.1. DNA Damage Response (DDR) and Cell Cycle Checkpoints

To ensure normal functioning and survival of a eukaryotic organism, it is extremely important to conserve and accurately transmit its genetic information from each cell to its daughter cells. However, cells are continuously exposed to endogenous and exogenous genotoxic agents that damage DNA, including oxidative stress. This, in turn, triggers an intricate signaling pathway known as the DNA Damage Response (DDR), which ultimately determines the fate of a cell following DNA damage. The DNA lesions are detected by several sensor proteins upstream of the DDR pathway, and this information is then relayed to a family of phosphoinositide 3-kinase related serine/threonine protein kinases (PIKKs), such as ataxia telangiectasia mutated (ATM), ATM and Rad3-related (ATR), and DNA-dependent protein kinase (DNA-PK). The PIKKs then convey these DNA damage signals to checkpoint control proteins.

ATR and ATM bind to the chromosomes at the site of DNA damage and trigger the activation of two other kinases, Chk1 and Chk2. This leads to activation of cell-cycle checkpoints that arrest the cell cycle briefly to provide time for cells to appropriately repair the DNA lesions. The cell cycle of a dividing eukaryotic cell involves four different phases: Gap1 (G1), Synthesis (S), Gap2 (G2), and Mitosis (M). However, metabolically active and viable cells that stop dividing enter a resting phase called Gap0 (G0). The G1, S, G2, and M phases in cells grown in culture last approximately 12, 6, 4, and 0.5 h, respectively [145]. DNA damage checkpoints, controlled by PIKKs, can be classified into the G1/S checkpoint, which prevents replication of damaged DNA, the intra-S phase checkpoint, which monitors cell cycle progression and decreases the rate of DNA synthesis following DNA damage, and the G2/M checkpoint, which allows suspension of the cell cycle prior to chromosome segregation. Once the damage has been repaired, checkpoint-arrested cells resume progression of the cell cycle. However, rapid accumulation of unrepaired DNA lesions at the checkpoint can induce permanent cell cycle arrest (senescence), or if the damage is too severe to be repaired, the cell may undergo programmed cell death (apoptosis). If the DNA damage is not repaired properly, it can cause errors during DNA replication, thus transmitting error-prone genetic information that lead to mutations.

While ATM and DNA-PK respond mainly to DSBs caused by ionizing radiation and radiomimetic drugs, ATR is activated by a broader spectrum of DNA damage, including stalled DNA replication forks, SSBs and bulky adducts induced by UV light and oxidative stress [146,147]. However, several studies have also determined that ATM can be activated by ATR and vice versa [148,149,150,151,152]. Jazayeri *et al.* [148] demonstrated that ATR is activated in response to DSBs in an ATM-dependent manner in the S and G2 phases of the cell cycle, while Adams *et al.* [149] reported that ATR is activated following ATM activation in response to ionizing radiation-induced DNA damage in the G1 and S cell cycle phases. On the other hand, Stiff *et al.* [152] showed that ATM is activated in an ATR-dependent manner in response to UV radiation and stalled replication forks.

#### 4.1.2. Dual Function of Tumor Suppressor p53

Tumor suppressor p53, a downstream target of ATM/ATR, plays an important role in mediating cellular responses to DNA damage. Under normal conditions, p53 levels are kept low in the nucleus by ubiquitination and proteosomal degradation. Following phosphorylation by ATM/ATR on serine-15, p53 is stabilized, which leads to its accumulation in the nucleus. This activated p53 may then trigger either cell cycle arrest and DNA repair, or alternatively, induce apoptosis if the DNA damage is too severe [153]. Transient cell cycle arrest at the G1/S and G2/M checkpoints is maintained by p53 through increased expression of the cyclin-dependent kinase (CDK) inhibitor, p21. Increased levels of p21 induce cell-cycle arrest by inhibiting the activity of the cyclin-CDK complex that regulates cell cycle progression [154]. However, under stress conditions, p21 can also be induced by a p53-independent mechanism [154]. Phosphorylated p53 may also up-regulate the expression of the pro-apoptotic factors Puma, Bax and Noxa, thereby inducing apoptosis. Moreover, activated p53 may also be involved in inducing cell senescence through induction of the CDK inhibitor p16 and tumor suppressor p19.

#### 4.1.3. DNA Damage Repair Mechanisms in Response to Oxidative Stress

**Figure 11:**
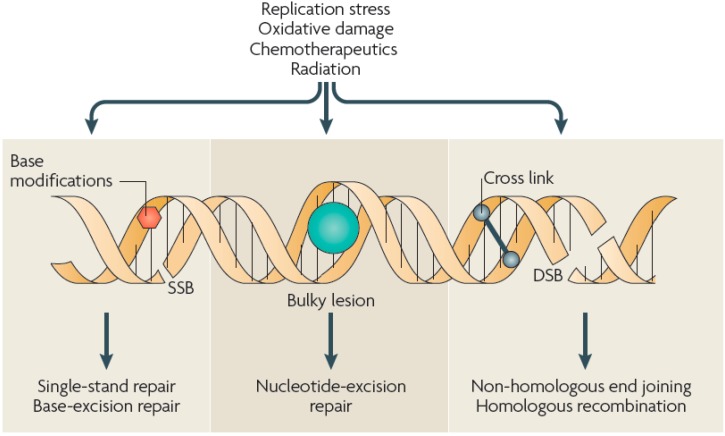
Types of DNA damage and repair. Various types of DNA damage can occur in cells as a result of endogenous agents, such as replication stress or free radicals from oxidative metabolism, and exogenous agents, such as ionizing or UV radiation and chemotherapeutics. These agents can cause SSBs or DSBs in the DNA, base modifications, helix-distorting bulky lesions or cross-links of DNA strands that are repaired by biochemically distinct DNA repair pathways. Adapted from [132] with permission from Macmillan Publishers, Ltd., 2009.

ROS have been implicated in a multitude of DNA modifications, including sugar and base modifications, DNA—protein cross-linking, and SSBs and DSBs. DSBs are the most severe form of DNA damage in eukaryotic cells, as inefficient repair may cause mutations or even cell death. Depending on the extent and type of DNA lesion and the stage of the cell cycle, various DNA damage repair systems are activated in eukaryotic cells, including base excision repair (BER) for SSBs, nucleotide excision repair (NER) for bulky adducts, non-homologous end joining (NHEJ) and homologous recombination (HR) for DSBs, and DNA mismatch repair (MMR) for correction of replication errors, such as base-pair mismatches and loops/bubbles arising from a series of mismatches (Figure 11) [132]. Whenever a homologous sequence, e.g., a sister chromatid, is available as a template, such as in the G2 and S phases of the cell cycle, a DSB is repaired by HR [134,135,136]. However, the absence of a homologous sequence in the G1 phase and the highly condensed chromatin structure in the G2/M phase decreases HR activity, and instead, recruits NHEJ for DSB repair [134,135,136]. NHEJ is active throughout the cell cycle, but is a highly error-prone repair mechanism. For instance, NHEJ repair of DNA cross-links induced by the drug cisplatin produced DSBs [155]. MMR plays an important role in removing mismatches during replication in the S phase.

Non-bulky base damages resulting from oxidation are removed primarily by BER [43,133]. One example of base damage that is widely studied is the oxidation of guanine to generate 8-oxo-guanine (8-oxo-G), which can cause mutations if unrepaired. This base damage is removed by short-patch BER via the action of a DNA glycosylase, 8-oxoguanine glycosylase (OGG1), which cleaves the *N*-glycosidic bond between the sugar-phosphate backbone and 8-oxo-G. However, this leaves an abasic/apurinic site (AP), which is still considered DNA damage, and is eventually processed by AP endonuclease that cleaves the phosphodiester bond at the AP site. DNA polymerase β then adds a single nucleotide (in this case guanine) to the AP site and DNA ligase to seal the nick [133]. If left unrepaired, 8-oxo-G can cause a mismatch in the nucleotide sequence during replication by base pairing with thymine rather than cytosine, resulting in T-A base pairing instead of G-C. While 8-oxo-G is regarded as the most common product of non-bulky oxidative damage to purine bases, thymine glycol is the most frequent product of damage to pyrimidine bases [156]. BER also repairs ROS-induced SSB that has a blocking residue at the 3ʹ terminal of the cleaved site [43,133]. This type of SSB is usually produced by the action of ROS on the sugar residues producing 3'-phosphoglycolate, 3'-phosphate or 3'-phosphoglycoaldehyde [157].

ROS have also been implicated in the formation of bulky adducts following direct reaction with DNA [158]. When a purine base forms a covalent bond with the 5'-carbon of the deoxyribose sugar of the same nucleoside and the closest pyrimidine base, these interactions produce two types of bulky adducts; purine cyclonucleosides and base-base intrastrand cross-links, respectively [158]. These lesions are mostly repaired by the NER pathway [159]. The damage repair begins with the unwinding of the DNA helix by XPB and XPD helicases. This is followed by dual excision by the endonucleases XPG and ERCC1/XPF of only one DNA strand at the 3' and 5' ends of the region containing the lesion, which removes the damaged nucleotides [160]. Using the complementary DNA strand as a template, the resulting gap is filled with new nucleotides by DNA polymerases δ or ε and associated replication factors. Finally, DNA ligase seals the nick in the new strand and thus completes the repair process [160]. Lesions such as cyclobutane pyrimidine dimers (CPDs) and pyrimidine-6,4-pyrimidone photoproducts ((6-4) photoproducts) induced in DNA following exposure to UV radiation and certain cytotoxic chemicals are also repaired by NER [161].

### 4.2. APP-Induced DNA Damage in Eukaryotic Cells and Associated Cellular Responses

*In vitro* and *in vivo* studies conducted in a variety of normal and cancer cell lines have demonstrated the efficacy of APPs in inducing a variety of dose-dependent effects ranging from cell proliferation to apoptosis [7,8,13,14,15,46,64,65,69,70,71,72,73,74,75,76,162,163]. Moreover, significant progress has been made over the years in understanding the mechanism of plasma-induced effects, specifically plasma-induced apoptosis in cancer cells. Many of these studies have reported DNA damage above a certain plasma dosage [46,65,69,70,71,73,74,75,162,163].

Phosphorylated H2AX, a well-known DNA damage marker, was employed by several plasma groups to detect DNA damage in eukaryotic cells following APP treatment. The phosphorylation of H2AX, a variant of the H2A family of histone protein, on the serine 139 residue referred to as γ-H2AX, is one of the earliest events that occurs in response to DNA damage [164]. Once phosphorylated, H2AX acts as a docking site for multiple DDR proteins that accumulate at the site of DNA damage and result in the formation of a nuclear foci that can be detected by several techniques, such as immunofluorescence microscopy, immunoblotting and flow cytometry. Additionally, flow cytometry is an excellent technique to study the changes in γ-H2AX intensity in relation to the distribution of cells in the various phases of the cell cycle. Interestingly, in addition to genotoxic agents, DNA fragmentation during apoptosis has also been shown to generate a large number of SSBs and DSBs that also result in extensive H2AX phosphorylation [146,165,166,167]. Hence, careful measurement and analysis with respect to morphology and kinetics of γ-H2AX should be conducted by plasma researchers to distinguish between γ-H2AX induced by direct DNA damage and that associated with apoptosis (which may also be induced by damage to other cellular components, such as the cell membrane), and also when making conclusions about the type of DNA lesion (SSB, DSB, bulky adducts, thymine dimer, *etc.*) based only on γ-H2AX staining [168].

Depending on the type of plasma source, dosage and cell type, plasma treatment has been shown to elicit multiple responses to the DNA damage induced, ranging from cell cycle arrest to DNA repair or apoptosis. An earlier study by Kim *et al.* [69] demonstrated that a surface type APP in air induced apoptosis in a dose-dependent manner in B16F10 melanoma cancer cells *in vitro*. At higher doses, they reported an increase in the DNA damage marker γ-H2AX, p53 tumor suppressor gene, and caspase-3, a downstream apoptosis effector, 3 h after plasma treatment. This was accompanied by an accumulation of cells in the sub-G1 phase of the cell cycle 24 h after plasma treatment, thus indicating DNA damage leading to apoptosis. Besides damage to DNA, this surface type APP also caused damage to the mitochondrial membrane and induced cytochrome C release. While not shown experimentally, they ascribed APP-induced DNA damage to the high concentration of O_3_ produced by their APP. While Kim *et al.* [69] attributed melanoma cell apoptosis to DNA damage, Leduc *et al.* [46] concluded that the DNA damage observed in their study might not be responsible for the cancer cell apoptosis observed. Leduc *et al.* [46] compared the effects of reactive species produced by a direct APP and an indirect APP, both ignited in He gas, on human adenocarcinoma HeLa cells *in vitro*. Immediately after plasma treatment, an increase in intracellular reactive species was observed in direct APP treated cells, as measured by an increase in the fluorescence intensity of the general ROS detection dye 2,7-dichlorodihydro-fluorescein diacetate (carboxy-H_2_DCFDA), while no increase was observed in indirect APP-treated cells. However, they attributed the lack of a fluorescence signal in indirect APP-treated cells to cell loss due to detachment. While DNA damage increased gradually up to 24 h post-treatment in both direct and indirect APP-treated cells, interestingly, caspase-3 increased only in the direct case. Apoptosis in HeLa cells was claimed to be induced by direct APP via oxidative stress and not by DNA damage, as no apoptosis was observed in indirect APP-treated cells, despite the fact that APP induced DNA damage.

Several research groups have designed DNA studies to explore the spatial extent [13,72] and penetration depths [73] of plasma effects on cellular systems. Han *et al.* [13,66] conducted a spatial distribution study to investigate the extent of DNA damage induced in SCC-25 oral cancer cells by an APP ignited in N_2_ gas. This type of study provides valuable information on the target area achieved by plasma treatment and hence, has significant clinical relevance with respect to cancer therapy. Interestingly, 3D mapping of the coverslips with cancer cells treated by plasma provided detailed information on the effective damage area and damage levels with respect to the plasma jet dimensions [66]. In general, for a relatively small plasma jet tip diameter of ~1 mm, a much larger effective area was observed even with 10 s of plasma treatment. A longer treatment time resulted in a wider effective area of DNA damage, as indicated by γ-H2AX staining; however, the number of cells with DNA damage decreased farther from the treatment center. Because the tip diameter is comparatively smaller than the effective damage area, they attributed the damaging effects to secondary interactions due to diffusion of reactive species and electrons produced by the N_2_ APP, which triggered complex chemical reactions that induced DNA damage in cells. In another study, Morales-Ramirez *et al.* [72] looked at the effect of axial distance from the source on DNA damage induced in mice leukocyte embedded in agarose using a radio-frequency APP generated by a He plasma needle. Employing a single-cell gel electrophoresis assay, also known as a comet assay, they showed exposure time-dependent DNA damage at a treatment distance of 0.5 cm, beginning with slight damage and proceeding to complete DNA fragmentation. However, complete fragmentation of the DNA close to the needle (0.1 cm) was observed for all treatment times. They also indicated that plasma-induced DNA damage was caused primarily by oxidative radicals rather than by UV light.

Plewa *et al.* [73] recently investigated plasma penetrative effects using multicellular tumor spheroids (MCTS) that mimic a microtumor in terms of 3D organization, and cell-cell and cell-environment interactions. They showed that an APP ignited in He gas dose-dependently inhibited the growth of colon carcinoma HCT116 MCTS (400 µm in diameter) and reduced the expression of the proliferation marker Ki67 [73]. They correlated these observations with a dose-dependent increase in γ-H2AX staining 4 h after plasma exposure that indicated DNA damage [73]. Interestingly, an ROS scavenger, *N*-acetyl cysteine, abrogated plasma-induced growth inhibition and DNA damage, and increased Ki67 staining, thus indicating that ROS are responsible for MCTS DNA damage and growth inhibition. The addition of conditioned media to MCTS also induced DNA damage, suggesting that reactive species produced by plasma in culture media play a major role in DNA damage.

In an attempt to characterize the mechanism of plasma interactions with cellular systems, several recent studies have reported the influence of APP on DNA damage and the subsequent effects on cell cycle progression [8,14,65,69,71,74,75,76,163]. Interestingly, many researchers have observed a G2/M checkpoint arrest following APP treatment [8,14,74,75,76,163].

APP treatment of U87MG human glioblastoma and colorectal carcinoma HCT-116 cells by Vandamme *et al.* [163] induced DNA damage 1 h after treatment that resulted in cell cycle arrest in the S and G2/M phases of the cell cycle. A comparison between directly treating cells in culture medium *vs.* adding treated medium to cells demonstrated that plasma-generated species in culture medium were responsible for inducing DNA damage that eventually led to cell cycle arrest and the induction of apoptosis in cancer cells. They also treated U87MG-bearing mice *in vivo*, and observed an S phase accumulation and apoptosis of tumor cells in the entire tumor volume, indicating either penetration of plasma effects or induction of ROS production inside the tissue. While it was not confirmed experimentally *in vivo*, they attributed the observed plasma effects to formation of DNA strand breaks. Another APP generated by a single electrode plasma jet device also induced cell cycle arrest at the G2/M cell cycle phase, leading to apoptosis of HepG2 human hepatocellular carcinoma cells *in vitro* [75]. Increased expression of p53 and p21 were also observed, with a corresponding decrease at the transcriptional level of two regulatory proteins, cyclin B1 and cdc2, which normally control G2 to M cell cycle progression.

**Figure 12:**
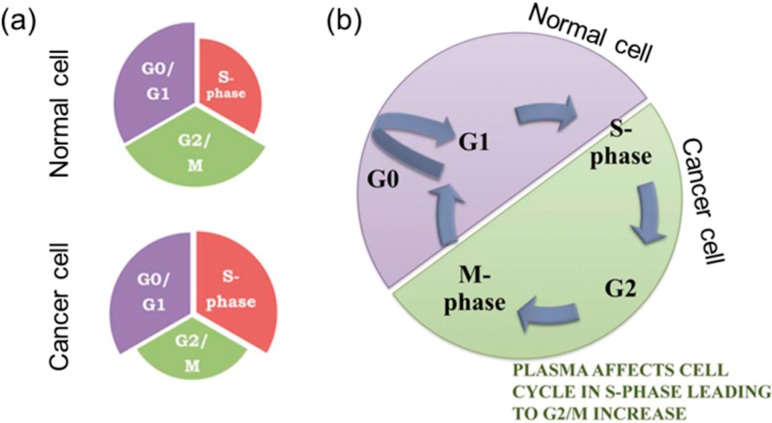
Schematic model of APP effects on the cell cycle. (**a**) Schematic representation showing a higher percentage of cancer cells than normal cells in the S-phase of the cell cycle; (**b**) Schematic representation of APP targeting the S-phase, leading to an increase of cells in the G2/M phase. Adapted from [74] with permission from Macmillan Publishers, Ltd., 2012.

Volotskova *et al.* [74] demonstrated that APP inhibited the cell cycle progression in mouse skin cancer cells (transformed keratinocytes) by accumulating them at the G2/M checkpoint ~24 h after plasma treatment. This observation correlated with an increase in DNA damage (γ-H2AX) and a decrease in DNA replication in the S-phase of the cell cycle. In short, they inferred that cancer cells are more sensitive to APP effects because a higher percentage of cells are in the S-phase (Figure 12). Moreover, analysis of the kinetics of H2AX phosphorylation suggested that the observed DNA damage was not DSBs.

While Vandamme *et al.* [163], Yan *et al.* [75], and Volotskova *et al.* [74] have observed G2/M cell cycle arrest in plasma-treated cancer cells, as mentioned above, Wende *et al.* [14] and Blackert *et al.* [8] made a similar observation in normal cells.

Wende *et al.* [14] showed a dose-dependent reduction in cell number and DNA synthesis of human HaCaT keratinocytes treated by an Ar APP (kINPen 09). They employed a single-cell gel electrophoresis assay in alkaline and neutral modes to identify DNA SSBs and DSBs, respectively. Interestingly, SSBs were detected immediately after treatment, declined within 4 h and returned to control levels after 24 h. In comparison, UV-B irradiation also immediately induced SSBs, but sustained higher than control levels for up to 48 h. In contrast, DSBs increased slowly over time and peaked at 6–12 h, which was attributed to apoptosis-associated DNA fragmentation and may not be due to direct DNA oxidation by the plasma, which also dropped to control levels within 24 h. APP treatment also resulted in a dose-dependent accumulation of the HaCaT keratinocytes in the G2/M phase of the cell cycle in response to the DNA damage observed. They concluded that APP induced transient and reversible DNA damage (SSBs) that slowed down cell cycle progression and ultimately, reduced DNA synthesis and resulted in decreased cell proliferation. These effects were attributed to intracellular ROS levels post-plasma treatment, which depended heavily on the ROS scavenging capacity of the treatment medium. In a similar study by Blackert *et al.* [8], HaCaT cells treated with a direct APP also reduced cell viability, while increasing both intracellular ROS levels and the accumulation of cells in the G2/M phase. Alkaline SCGE showed a dose-dependent increase in DNA damage within 1 h which, except at high doses, returned to control values at 24 h. These effects were diminished when the treatment medium was replaced immediately after plasma treatment, thus indicating the role of long-living reactive species produced by the interaction of plasma ROS with medium components such as amino acids, vitamins, *etc.*, that induced DNA damage and cell-cycle arrest.

In order to further elucidate the molecular mechanism associated with APP-induced DNA damage and cell-cycle arrest, additional studies were conducted to identify which pathway, ATM or ATR, triggered the DNA damage response in eukaryotic cells in response to plasma treatment [7,65,70]. These studies also attempted to identify the role of tumor suppressor p53 in determining cell fate following plasma exposure [65,71].

**Figure 13:**
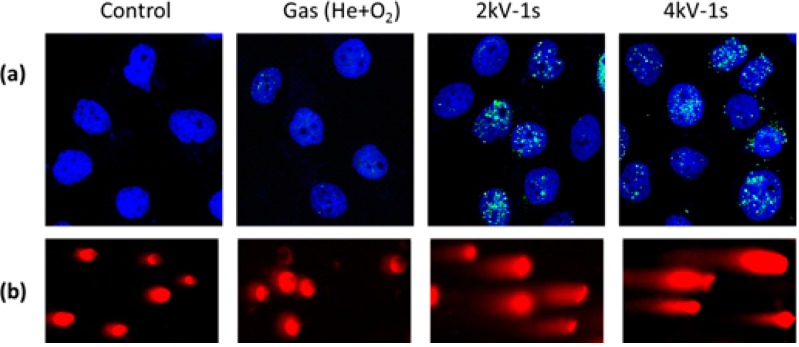
Effect of APP on DNA damage in SCC25 oral cancer cells. (**a**) Immunocytochemistry of γ-H2AX; SCC25 cells treated with 2 and 4 kV of NTP for 1 s showed increased γ-H2AX staining 24 h after APP treatment, indicating DNA damage; (**b**) Comet assay; cells containing DNA breaks (with long tails) were observed by fluorescence microscopy and quantified according to tail length, representing the extent of DNA damage. Adapted from [65] with permission from Elsevier, Inc., 2014.

A recent study by Chang *et al.* [65] observed that a spray-type APP ignited in a He/O_2_ mixture induced DNA damage and apoptosis in both wild-type (SCC25) and p53-mutated (MSK QLL1, SCC1843 and SCC15) oral squamous carcinoma cells (OSCC). An increase in γ-H2AX foci in SCC25 cells was observed 24 h after plasma treatment (Figure 13a). A comet assay revealed cells containing long tails, indicating breaks in DNA (Figure 13b). However, the plasma triggered a sub-G1 cell cycle arrest only in wild-type SCC25 cells. Interestingly, they also detected increased expression of ATM, p21 and p53 in SCC25 cells, indicating activation of the ATM/p53 pathway in response to DNA damage and leading to cell cycle arrest and apoptosis of SCC-25 cancer cells. Additional investigation is required, as it was found that in addition to ATM activation, plasma also induced ATR phosphorylation. This study was supported by the findings of Kim *et al.* [70], who detected increased levels of phospho-p53 and γ-H2AX in N_2_ APP-treated ATM-complemented YZ5 cells, but not in ATM-deficient S7 cells. Furthermore, they observed increased H2AX phosphorylation in HCT15 human colon cancer cells with wild-type Chk2 compared to kinase-dead Chk2. Hence, they concluded that APP-induced DNA damage that activated the ATM-Chk2 pathway and p53 tumor suppressor protein, leading to apoptosis.

In contrast, Kalghatgi *et al.* [7] and Lazovic *et al.* [77] determined that plasma-induced phosphorylation of H2AX is ATR-dependent and not ATM-dependent. In their dose-dependent study of APP treatment of MCF10A human breast epithelial cells *in vitro*, Kalghatgi *et al.* [7] observed cell proliferation at low doses and apoptosis at high doses. They attributed these dose-dependent effects to the formation of intracellular ROS. They also demonstrated that neutral plasma ROS and not UV radiation or charged particles were instrumental in phosphorylation of H2AX, likely due to the formation of organic peroxides in the culture medium. However, there were no bulky adducts or formation of thymine dimer. Hence, they presumed that the increase observed in γ-H2AX staining may have been due to formation of DNA SSBs or replication arrest. In addition, the same group demonstrated that APP induced lipid peroxidation in MCF10A cells; however, they concluded that plasma-induced DNA damage is not mediated via plasma-induced lipid peroxidation [67]. They also demonstrated that the DNA damage observed was not mediated by plasma-produced ozone [68]. Lazovic *et al.* [77] showed that a capacitively-coupled APP ignited in He directly caused SSBs and bulky lesions in fibroblasts, but also induced DSBs as a consequence of DNA repair. They also observed small γ-H2AX foci typical of ATR-induced H2AX phosphorylation following APP exposures.

Ma *et al.* [71] conducted an extensive study on 17 mammalian cell lines to investigate the anti-tumorigenic effects of APP generated in He gas. at the same treatment dose, APP selectively induced DNA damage and apoptosis in cancer cells compared to normal cells and stem cells. Interestingly, for the same treatment conditions, p53-deficient (*p53*^−/−^) cancer cells showed hypersensitivity to plasma by comparison to p53-proficient (*p53*^+/+^) cancer cells. The apoptotic effect of plasma was greater for p53-deficient cells, while artificial p53 expression in p53-deficient cells decreased sensitivity to plasma. They concluded that, in p53-proficient cells, plasma-induced DNA damage activated p53 and the downstream apoptotic factors Puma and Bax, causing a G1 cell cycle delay that eventually led to cell apoptosis. Meanwhile, in p53-deficient cells, plasma-induced DNA damage accelerated apoptosis independent of the p53 pathway and without a G1 delay. The presence of ROS scavengers, *N*-acetyl cysteine and sodium pyruvate, abrogated DNA damage and apoptosis, indicating that ROS generated by APP are crucial in inducing DNA damage and apoptosis. Moreover, APP also induced DNA damage and apoptosis in chemotherapeutic drug-resistant cancer cell lines.

Poly(ADP-ribose)polymerase-1 (PARP-1) is a nuclear enzyme activated in response to DNA damage, primarily SSBs, to initiate DNA damage repair [169]. However, the proteolytic cleavage of 116 kDa PARP-1 by caspases-3 and -7 to 85 and 24 kDa fragments is a characteristic event of apoptosis [170,171]. Hence, measurement of PARP-1 cleavage indicates DNA damage leading to apoptosis. A N_2_ APP generated with a micronozzle array induced DNA damage and increased the apoptosis marker proteins, caspase-3 and poly(ADP-ribose) polymerase (PARP) in human embryonic kidney 293T cells [70]. In another approach, Ar microwave plasma treatment of skin cells (NIH3T3 mouse fibroblasts and HaCaT keratinocytes) by Choi *et al.* [76] showed no induction of p53 and PARP cleavage, thus indicating the absence of DNA damage-induced apoptosis. However, they observed cell cycle arrest in the G2 phase and a p53-independent increase in p21, but no cell death [76]. The cell cycle arrest was abrogated upon replacement with fresh media immediately after treatment, indicating the role of plasma-produced components in the cell culture medium.

A few studies have also compared DNA damage induced by plasma with that induced by UV [64], X-ray [162] and gamma [77] radiation. A study conducted by Brun *et al.* [64] demonstrated a decrease in microbial load that did not affect the viability of ocular cells (keratinocytes and conjunctival fibroblasts) after treatment with an APP. However, they observed a transient increase in the expression of the oxidized base, *i.e.*, 8-oxodeoxyguanosine (8-OHdG) in plasma-treated keratinocytes, which returned to control levels within 24 h. Furthermore, they observed an increase in the expression of OGG1, a DNA glycosylase enzyme involved in the removal of mutagenic 8-OHdG by BER. APP treatment of human cornea *ex vivo* showed an increase in OGG1 mRNA and protein levels; however, no thymine dimerization was observed in the nuclei of APP treated corneal tissue. By comparison, UV treatment of corneal tissue *ex vivo* induced significant formation of thymine dimers.

Graham *et al.* [162] investigated the response of MDA-MB-231 human breast cancer cells exposed to an APP generated directly in the growth medium. A linear dependence between the average number of DNA damage foci, detected by γ-H2AX staining, and the number of plasma pulses applied was observed based on a Poisson damage distribution curve. Correspondingly, a decrease in the viability of cells was also observed. Interestingly, they observed a similar damage pattern on the same cell line exposed to 160 keV X-ray irradiation, and deduced that 100 plasma pulses would cause similar DNA damage as 1 Gy of X-ray irradiation in MDA-MB-231 breast cancer cells. They concluded that APP-liquid interaction and radiolysis follow similar liquid chemistry, ultimately leading to their biological effects.

Lazovic *et al.* [77] compared the effects of APP and gamma (Co^60^ γ-ray) irradiation on fibroblasts by measuring DSBs via γ-H2AX staining at various times following treatment. Interestingly, maximum DSB induction was detected 30 min and 2 h after gamma irradiation and APP treatment, respectively. In the case of gamma irradiation, the number of γ-H2AX foci increased linearly with treatment dose, while for APP treatment, it increased with both treatment time and power. Comparing the number of γ-H2AX foci per cell after gamma and plasma treatment, they also obtained the effective doses of plasma irradiation comparable to gamma irradiation. As mentioned before, they demonstrated that APP-induced H2AX phosphorylation was ATR-dependent, while it was ATM-dependent in the case of gamma irradiation. In addition, they observed heavily damaged nuclei typically caused by charged particles in APP-treated samples.

Because *in vitro* studies showed induction of apoptosis via DNA damage in both cancer and normal cells in a dose-dependent manner after APP treatments, follow-up *ex vivo* [15] and *in vivo* [9] studies were conducted to investigate those processes under more realistic conditions.

Isbary *et al.* [15] treated human skin with two APP devices based on surface microdischarge (SMD) technology *ex vivo* and showed, over shorter treatment times, significantly higher, as well as significantly lower, DNA damage in plasma-treated skin compared to control skin samples. Higher DNA damage was observed with a treatment time of 120 s compared to the control. Interestingly, they also observed that a higher initial cell load provided a protective effect from DNA damage for other cells. However, the damage was not localized to the higher cell layers, thereby warranting further investigation into the penetration of plasma effects into deeper cell layers.

A preliminary toxicity study conducted *in vivo* by Wu *et al.* [9] investigated the effects of a direct APP on DNA damage in intact and wounded skin of Yorkshire pigs. They observed significant accumulation of γ-H2AX only in skin exposed to more than a 5 min treatment at a power setting of 0.17 W/cm^2^, while lower treatment times showed no H2AX phosphorylation, indicating the absence of DNA damage [9]. These studies also concluded that there were dose-dependent effects for DNA damage induction by plasma treatment.

In order to understand APP effects on DNA damage, repair and recovery in mammalian systems, several groups have conducted experiments on a model microbe for eukaryotic cells, *Saccharomyces cerevisiae* (budding yeast). A recent study by Lee *et al.* [78] reported the induction of DSBs in yeast by an APP ignited in air, leading to loss of cell viability in a dose-dependent manner. Interestingly, these effects were enhanced in *rad51* mutants lacking the Rad51 protein required for the repair of DNA DSB via homologous recombination. They also observed, that compared to wild-type yeast cells, cells deficient in other HR proteins, such as Rad52 and Mec1 (yeast analog of human ATR), were also more susceptible to air plasma treatment. Because the antioxidant *N*-acetyl cysteine and NO scavenger c-PTIO failed to rescue the cells from cell death, they concluded that DSBs induced by plasma do not occur via ROS/RNS generation.

Ryu *et al.* [79] observed differential inactivation of yeast treated with an Ar APP in various liquid environments (water, saline and Yeast extract, Peptone, Dextrose (YPD). The highest inactivation of yeast cells was obtained in water and the lowest was in YPD. Agarose gel electrophoresis analysis of genomic DNA extracted from treated yeast cells showed significant DNA damage after plasma exposure in saline and water, but no damage in YPD. Besides DNA damage, plasma treatment in the presence of water and saline also induced lipid peroxidation and damage to proteins. Higher levels of ^●^OH radicals were also detected in plasma-treated water and saline compared to YPD. These results indicate a crucial role of the liquid environment of microbes in determining the outcome following exposure to plasma.

### 4.3. APP-induced DNA Damage in Prokaryotic Cells and Associated Response

In order to cope with various types of DNA damage, bacteria possess a novel mechanism known as the SOS response. There are two key proteins that control the SOS response: LexA and RecA. In the absence of DNA damage, the repressor protein LexA binds to the SOS box (a 20 base pair regulon consisting of *lexA* and *recA* genes), thereby switching off the SOS response, while the inducer protein RecA scans for DNA damage. In the event of DNA damage, RecA binds to SSBs and cleaves LexA resulting in activation of the SOS response, which leads to up-regulation of SOS genes. The first genes induced are the *uvr* genes involved in the NER pathway, followed by *lexA* and *recA* genes. If the DNA damage is too severe, genes encoding the highly error prone repair DNA polymerases *polB*, *dinB*, *umuC* and *umuD* are activated.

Over the years, several groups have demonstrated rapid inactivation of gram-positive and gram-negative bacteria, including both vegetative cells and spores, by low temperature APPs [57,58,80,81,82,83,84,85]. Several of these studies investigating the mechanism of plasma inactivation of microbes reported DNA damage as one of the detrimental effects induced by APP. A brief summary of these studies with a particular focus on DNA damage is presented in this section.

Lu *et al.* [86] observed that the extent of genomic DNA damage following exposure to APP depended on the type of bacteria (gram-positive/gram-negative) and treatment time. PCR amplification of DNA extracted from treated bacteria showed that a short treatment time (5 s) had no effect on DNA damage, while a 30 s exposure to APP induced significant DNA damage in *L. monocytogenes*, which correlated with its higher inactivation. Besides DNA damage, they also observed significant damage to the membrane in *E. coli* compared to *L. monocytogenes* as indicated by leakage of intracellular components. They concluded that the different damage patterns observed in the two bacterial strains were likely due to the difference in their membrane structure and resistance to damaging agents.

Joshi *et al.* [80] reported dose- and concentration-dependent inactivation of *E. coli* treated with a direct APP. Further investigation revealed depolarization of the bacterial cell membrane, as well as lipid peroxidation that lead to loss of membrane integrity. They also measured significant levels of the DNA damage marker, 8-OHdG. They concluded that the membrane damage induced by plasma propagated into the cell, causing DNA damage, and finally, *E. coli* cell death. On the other hand, Kvam *et al.* [81] observed only a minor increase in damage to DNA and protein following direct APP treatment. Hence, they concluded that DNA damage and oxidative stress were not responsible for the observed inactivation of multidrug resistant microbes.

Tseng *et al.* [82] reported inactivation of spores of *Bacillus* and *Clostridium* species treated with a He APP. Interestingly, the inactivation of *Bacillus subtilis* vegetative cells was achieved with a lower treatment time than *Bacillus subtilis* spores. However, no visible degradation of DNA extracted from vegetative cells and spores that were subjected to 20 min of plasma treatment prior to extraction was observed using a gel electrophoresis technique. On the other hand, naked DNA extracted from the vegetative cells and spores showed damage after 5 min of plasma treatment, with severe fragmentation after 20 min treatment. Hence, they concluded that plasma-induced DNA damage may not be the reason for the inactivation of vegetative cells and spores. In fact, they attributed the inactivation to spore coat leakage, indicated by an increase in the coat chemical, dipicolinic acid (DPA).

To study the differential regulation of genes in microbes in response to plasma treatment, several groups have conducted extensive transcriptome analysis using a DNA microarray [83,84,85]. Mols *et al.* [83] achieved 99.9% inactivation of *B. cereus* vegetative cells on surfaces within 5 min treatment of an APP ignited in N_2_ gas. However, they observed that the nucleotide excision repair genes involved in the SOS response, such as *uvrA* and *uvrB*, were not affected following plasma treatment. In contrast, Winter *et al.* [85] observed the up-regulation of *uvrA*, *uvrB* and *uvrC* in gram-positive *B. subtilis* 168 cells in liquid treated with an Ar APP. Moreover, the induction of *recA*, *lexA*, *dinB*, *yhaZ* and *ydgG* genes in addition to *uvrABC* genes led them to conclude that plasma-induced DNA damage is primarily due to UV. Supporting the results of Winter *et al.*, Sharma *et al.* [84] also observed up-regulation of *uvrA* and *uvrB* genes in plasma treated gram negative *E. coli*, indicating induction of DNA damage following plasma exposure. However, the absence of the genes *uvrC*, *uvrD*, and *polA* involved in the NER pathway indicated incomplete induction of DNA damage repair. They also suggested a synergistic involvement of plasma-produced UV and reactive species in inducing DNA damage and inactivation of *E. coli*.

Reporter gene studies conducted by Lackmann *et al.* [58] observed thymine dimer formation in *E. coli* DH5α cells treated with an APP jet. They identified VUV radiation, and not particles, emitted from the APP as responsible for the dimerization observed. Another study conducted by the same group monitored gene expression using various reporter gene fusions and observed UV-induced DNA damage (monitored using *recA*) in *B. subtilis* vegetative cells treated in liquid [57]. However, they concluded that the DNA damage observed was relatively less significant compared to protein damage and oxidative stress in inactivating *B. subtilis* under their experimental conditions.

Employing bacteriophages as surrogates for viruses, several groups have also investigated the potential of APPs in inactivating viruses [87,88]. Venezia *et al.* [87] observed that APP treatment of temperate λ bacteriophage C-17 and lytic bacteriophages for 10 min rendered them inactive. Interestingly, they observed damage to the cell wall, but no damage to the DNA. Yasuda *et al.* [88] observed rapid inactivation of λ phages within 20 s of APP treatment. Even though they observed increased DNA damage with increased plasma treatment, they concluded that the observed inactivation of bacteriophages is not due to DNA damage, but due to damage to coat proteins [88].

Prokaryotes responded to irreparable DNA damage differently from multicellular eukaryotes. In multicellular organisms, any DNA damage left unrepaired can cause mutations leading to uncontrolled proliferation that is detrimental to the organism. As the survival of the whole organism is more important than the survival of individual cells, the response to unrepaired DNA damage is permanent cell cycle arrest or apoptosis. However, in the case of prokaryotes, each cell is an organism whose survival is dependent on continued cell division, and therefore, continuale division with unrepaired DNA damage is advantageous regardless of the risks. Interestingly, several groups have also reported mutation in microbes treated with APP. While Wang *et al.* [89] reported mutation in *Streptomyces avermitilis* spores following exposure to a He APP, Fang *et al.* [90] induced mutation in a filamentous cyanobacterium, *Spirulina platensis*, using an APP ignited in air. While these mutations were beneficial in the studies reported above, they highlight the potential mutagenic effects of APP treatment that should be investigated carefully.

## 5. Conclusions

**Figure 14:**
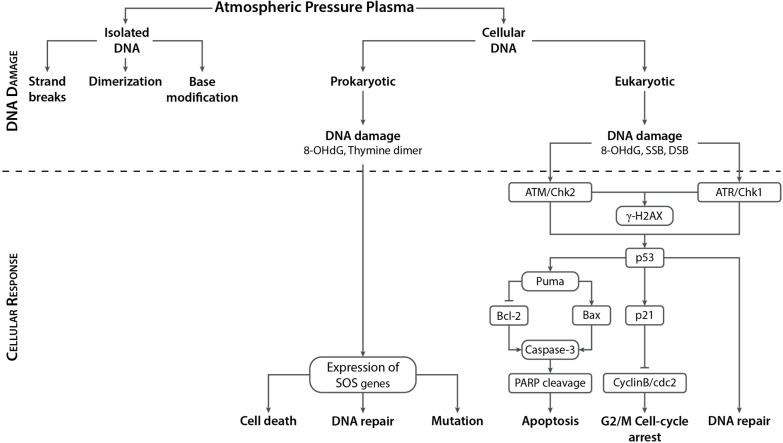
Summary of APP effects on isolated and cellular DNA. Studies on isolated DNA have shown that APP induces strand breaks, dimerization and base modifications. In prokaryotic cells, APP induced thymine dimerization and oxidation of DNA bases leading to formation of 8-OHdG. Depending on the extent of damage, DNA damage repair or cell death was initiated. However, mutation in response to DNA damage was also reported in prokaryotic cells. In response to DNA damage in eukaryotic cells, ATM and/or ATR were activated, which then phosphorylated p53. This in turn activated p21 and subsequent DNA repair mechanisms. Increased levels of p21 induced cell-cycle arrest by inhibiting the activity of the cyclinB-cdc2 complex leading to G2/M cell cycle arrest. In the event of irreparable DNA damage, p53 activation also caused activation of pro-apoptotic factors, such as Puma, Bax and caspase-3, which lead to apoptosis.

Advancements and developments in plasma medicine and its successful applications require continual research in parallel with clinical trials in order to enhance our knowledge about the exact physical, chemical and biological processes operating at the molecular level. Over the last fifteen years, great effort has been made to understand the effects of APPs, which can be used to further develop plasma sources to deliver precise doses and a specific type of ROS/RNS for a variety of biomedical applications. A summary of the APP effects observed on isolated and cellular DNA is shown in Figure 14.

This review emphasized the importance of understanding the underlying mechanisms regarding plasma-induced damage to DNA. It also revealed, through the sometimes conflicting results, the challenging nature of determining its effects. Due to the inherent and intrinsic complexity of plasma interactions with DNA, such interactions need to be investigated through different scientific approaches at various scales, starting from small segments of DNA to DNA in a cellular environment to enable their true scientific benefit to be understood. Their potential usage and impact warrants this further study.
